# Oxytocin blocks enhanced motivation for alcohol in alcohol dependence and blocks alcohol effects on GABAergic transmission in the central amygdala

**DOI:** 10.1371/journal.pbio.2006421

**Published:** 2019-04-16

**Authors:** Brendan J. Tunstall, Dean Kirson, Lia J. Zallar, Sam A. McConnell, Janaina C. M. Vendruscolo, Chelsea P. Ho, Christopher S. Oleata, Sophia Khom, Maurice Manning, Mary R. Lee, Lorenzo Leggio, George F. Koob, Marisa Roberto, Leandro F. Vendruscolo

**Affiliations:** 1 Neurobiology of Addiction Section, Intramural Research Program, National Institute on Drug Abuse, National Institutes of Health, Baltimore, Maryland, United States of America; 2 Department of Neuroscience, Alcohol Research Center, The Scripps Research Institute, La Jolla, California, United States of America; 3 Section on Clinical Psychoneuroendocrinology and Neuropsychopharmacology, National Institute on Alcohol Abuse and Alcoholism Division of Intramural Clinical and Biological Research and National Institute on Drug Abuse Intramural Research Program, National Institutes of Health, Bethesda, Maryland, United States of America; 4 Department of Cancer Biology, University of Toledo College of Medicine and Life Sciences, Toledo, Ohio, United States of America; 5 Center for Alcohol and Addiction Studies, Department of Behavioral and Social Sciences, Brown University, Providence, Rhode Island, United States of America; 6 Medication Development Program, National Institute on Drug Abuse Intramural Research Program, Baltimore, Maryland, United States of America; UCSD, United States of America

## Abstract

Oxytocin administration has been reported to decrease consumption, withdrawal, and drug-seeking associated with several drugs of abuse and thus represents a promising pharmacological approach to treat drug addiction. We used an established rat model of alcohol dependence to investigate oxytocin’s effects on dependence-induced alcohol drinking, enhanced motivation for alcohol, and altered GABAergic transmission in the central nucleus of the amygdala (CeA). Intraperitoneal oxytocin administration blocked escalated alcohol drinking and the enhanced motivation for alcohol in alcohol-dependent but not nondependent rats. Intranasal oxytocin delivery fully replicated these effects. Intraperitoneal administration had minor but significant effects of reducing locomotion and intake of non-alcoholic palatable solutions, whereas intranasal oxytocin administration did not. In dependent rats, intracerebroventricular administration of oxytocin or the oxytocin receptor agonist PF-06655075, which does not cross the blood-brain barrier (i.e., it would not diffuse to the periphery), but not systemic administration of PF-06655075 (i.e., it would not reach the brain), decreased alcohol drinking. Administration of a peripherally restricted oxytocin receptor antagonist did not reverse the effect of intranasal oxytocin on alcohol drinking. *Ex vivo* electrophysiological recordings from CeA neurons indicated that oxytocin decreases evoked GABA transmission in nondependent but not in dependent rats, whereas oxytocin decreased the amplitude of spontaneous GABAergic responses in both groups. Oxytocin blocked the facilitatory effects of acute alcohol on GABA release in the CeA of dependent but not nondependent rats. Together, these results provide converging evidence that oxytocin specifically and selectively blocks the enhanced motivation for alcohol drinking that develops in alcohol dependence likely via a central mechanism that may result from altered oxytocin effects on CeA GABA transmission in alcohol dependence. Neuroadaptations in endogenous oxytocin signaling may provide a mechanism to further our understanding of alcohol use disorder.

## Author summary

Alcohol use disorder is characterized by a reduction in reward function and gain in stress function. The neuropeptide oxytocin is involved in the regulation of both reward and stress systems. We tested the hypothesis that oxytocin administration could normalize the dysregulations that occur in alcohol dependence and thereby reduce alcohol drinking in dependent rats. We demonstrated that oxytocin administered systemically, intranasally, or into the brain blocked the enhanced drinking exhibited by alcohol-dependent rats. These effects were demonstrated to be centrally rather than peripherally mediated. Oxytocin blocked this enhanced alcohol drinking at doses that did not alter non–alcohol-related behaviors or alcohol drinking in nondependent rats, suggesting the effect was specific to alcohol drinking in alcohol dependence. *Ex vivo* electrophysiological recordings in the central nucleus of the amygdala (CeA; a key brain region in the network of dysregulations induced by alcohol dependence) indicated that oxytocin blocked the facilitatory effects of alcohol on inhibitory signaling in dependent but not nondependent rats. These results provide compelling evidence that dysregulation in the endogenous oxytocin system is of functional relevance to a mechanistic understanding of alcohol use disorder.

## Introduction

Alcohol use disorder is a global public health issue; 6% of the world’s population is subject to morbidity and mortality from alcohol [1]. Alcohol dependence (reflecting dysfunction equivalent to moderate to severe alcohol use disorder) is a chronic, relapsing disorder that develops as a result of neuroadaptations in brain reward, stress, and executive function systems controlled by neurocircuits that involve the basal ganglia, extended amygdala, and prefrontal cortex, respectively [2]. In alcohol dependence, the motivation to seek and consume alcohol is driven by reward hypofunction and stress sensitization. Both of these processes contribute to a negative emotional state that drives enhanced motivation for alcohol drinking via negative reinforcement [3,4].

Oxytocin has been hypothesized to have an important role in social behavior, fear and anxiety, and learning and memory [5]. Recent optogenetic and neuroanatomical mapping studies have advanced our understanding of the complexity of oxytocin’s neurocircuitry and the role of signaling by the endogenous oxytocin system in various behaviors [6]. Oxytocin modulates stress and reward function and has long been suggested as a putative treatment for addiction [7]. Reports that oxytocin administration can reduce drug consumption, withdrawal, and relapse associated with various drugs of abuse in preclinical models has greatly enhanced the interest in this area [8,9]. Indeed, oxytocin decreased economic demand for stimulants [10], reinstatement of stimulant seeking [11,12], opioid tolerance and withdrawal [13], and development of alcohol tolerance [7]. Particularly relevant for treating alcohol dependence, oxytocin has an anti-stress and anti-anxiety profile [14]. Preliminary clinical studies have demonstrated that intranasal oxytocin administration reduced alcohol-withdrawal–related anxiety and alcohol craving in alcohol-dependent patients in early abstinence [15], reduced alcohol craving in anxious alcohol-dependent individuals [16], and reduced neural response to alcohol cues in heavy social drinkers [17]. Oxytocin decreased drinking in nondependent rats [18,19], binge drinking in mice [20,21], and cue-induced reinstatement in postdependent rats [17]. However, oxytocin’s effects on alcohol drinking and motivation for alcohol in currently alcohol-dependent rats remains to be determined. Therefore, in the present study, we used a model of chronic-intermittent exposure to alcohol vapor to induce alcohol dependence and enhanced motivation for alcohol. This model has excellent predictive validity, has allowed dissociation of the neurobiology underlying alcohol dependent versus nondependent behavior, and has been used to determine the likely contribution of a range of neurobiological systems to alcohol use disorder [3,39].

Oxytocin has been detected in the brain following both intraperitoneal and intranasal administration in mice and rats [22–24]. Oxytocin receptors are found in the brain and in various peripheral tissues, where binding mediates oxytocin’s classic role in lactation, parturition, and sexual reflexes in males and females [25]. There are several other oxytocin binding sites in the periphery, including the gut, heart, vascular system, and vagus nerve, which all may contribute to behavioral effects of oxytocin, including inhibition of appetite and fear [25,26]. The central versus peripheral contributions to oxytocin’s putative effects on alcohol drinking also remain to be determined.

Oxytocin receptors are found in many brain regions relevant for alcohol dependence, such as the extended amygdala in rats and humans, including the central nucleus of the amygdala (CeA) [27,28]. The CeA is well described as a key structure involved in the transition to drug and alcohol dependence [2,29]. Silencing of an alcohol-withdrawal–activated CeA neuronal ensemble blocked the enhanced drinking associated with alcohol dependence [30]. The majority of CeA neurons are GABAergic, and increased GABA signaling in the CeA is linked to alcohol dependence. Intra-CeA infusion of a GABA_A_ receptor agonist decreased alcohol drinking in alcohol-dependent rats [31]. We previously demonstrated that spontaneous and evoked GABA transmission is increased in CeA of alcohol-dependent rats [32]. Both pro- and anti-stress neuropeptides interact with CeA GABA signaling and bidirectionally modulate alcohol drinking. Oxytocin has been reported to interact with GABAergic signaling and may block the acute effects of alcohol via a direct action on GABA_A_ receptors [33]. However, the physiological effects of CeA oxytocin in alcohol dependence are currently unknown.

Therefore, in the present study, we tested the hypothesis that oxytocin decreases alcohol drinking and motivation for alcohol specifically in alcohol-dependent rats via central and not peripheral actions. Furthermore, we hypothesized that alcohol dependence would alter how oxytocin modulates GABA signaling in the CeA.

## Materials and methods

### Ethics statement

For all behavioral and electrophysiological experiments involving anesthesia, isoflurane inhalation was used. For electrophysiological experiments, anesthesia was followed by rapid decapitation to allow brain slice preparation. All behavioral studies were approved by the Institutional Animal Care and Use Committee (ACUC) of the National Institute on Drug Abuse Intramural Research Program. All electrophysiological procedures were approved by the IACUC of The Scripps Research Institute. All procedures were conducted according to the National Institutes of Health Guide for the Care and Use of Laboratory Animals (8th edition).

### Animals

Rats used in behavioral (male Wistar, *n* = 86; Charles River, Kingston, NY) and electrophysiology (male Sprague-Dawley, *n* = 87; Charles River, Raleigh, NC) experiments weighed 225 to 275 g upon arrival, were group-housed (2 to 3 per cage) in standard plastic cages in a temperature- and humidity-controlled room, and were maintained under a reverse 12 h/12 h light/dark cycle with food and water available ad libitum except during behavioral testing.

### Drugs

For behavioral experiments, alcohol (ethanol; Warner Graham, Cockeysville, MD) was dissolved in tap water. Oxytocin (ChemPep Inc., Wellington, FL) was dissolved in 0.9% saline. For peripheral administration, the non–blood-brain barrier-penetrant oxytocin receptor agonist PF-06655075 was initially dissolved in 10% (v/v) Self Emulsifying Drug Delivery System that consisted of 3:4:3 Miglyol 812:Cremophor RH40:Capmul MCM (v/v/v). The emulsion was completed with 90% (v/v) 50 mM aqueous phosphate buffer (pH 7.4). For intracerebroventricular administration, PF-06655075 was initially dissolved in 5% (v/v) dimethyl sulfoxide, then combined with 5% (v/v) polyethylene glycol 300, and completed with 90% (v/v) saline. PF-06655075 and the vehicle used for its peripheral administration were kindly provided by Pfizer. The non–blood-brain barrier penetrant antagonist L-371,257 (Tocris Bioscience, Ellisville, MO) was prepared in a vehicle of 5% (v/v) dimethyl sulfoxide, 5% (v/v) Cremophor, and 90% (v/v) sterile water.

For electrophysiology experiments, oxytocin and the selective vasopressin receptor 1A antagonist (d(CH2)5,Tyr(Me)2,Arg8)-Vasopressin (TMA) [34] were obtained from Tocris (Ellisville, MO). The selective oxytocin receptor antagonist desGly-NH2-d(CH2)5[D-Tyr2,Thr4]OVT (OTA) was provided by Dr. Maurice Manning (University of Toledo, OH) [34]. CGP 55845A, DL-2-amino-5-phosphonovalerate (DL-AP5), and 6,7-dinitroquinoxaline-2,3-dione (DNQX) were obtained from Tocris (Ellisville, MO). Tetrodotoxin (TTX) was purchased from Biotium (Hayward, CA). Alcohol was purchased from Remet (La Mirada, CA, USA). All drugs were dissolved in artificial cerebrospinal fluid (aCSF).

### Operant alcohol self-administration and alcohol vapor exposure

Oral alcohol self-administration experiments were conducted in standard operant chambers (Med Associates, St. Albans, VT) fitted with 2 retractable levers and a dual-cup liquid receptacle. After initial training, as previously described [29,35], the animals were allowed to lever press for alcohol (10%, w/v; 0.1 ml) and water (0.1 ml) on separate levers according to a concurrent fixed-ratio 1 (FR1) schedule of reinforcement (each lever press resulted in fluid delivery) in 30-min operant sessions. After each training session, the liquid receptacle and surrounding area were inspected to confirm the consumption of earned reinforcers. After response acquisition, rats were split into 2 groups matched by their alcohol consumption. For the remainder of these experiments, rats in the nondependent group were exposed to air without alcohol, whereas rats in the dependent group were exposed to alcohol vapor in daily cycles designed to cause intoxication (14 h vapor “on”; 200 mg/dl target blood alcohol levels) and withdrawal (10 h vapor “off”) to induce alcohol dependence, as previously described [29,35]. Dependence is characterized by somatic and motivational signs of withdrawal that include increased anxiety-like behavior, reward deficits, and enhanced motivation to self-administration of alcohol [36–39]. In all operant alcohol self-administration experiments, steady baseline consumption of alcohol was established in dependent and nondependent rats before pharmacological testing began. Operant alcohol self-administration sessions including pharmacological tests were conducted 2 to 3 sessions per week (never on consecutive days to minimize potential carry-over effects) during the 10 h “off” period, 6 to 8 h into withdrawal.

### Effect of intraperitoneal oxytocin on alcohol intake (FR1) and motivation (progressive ratio)

Intraperitoneal oxytocin (0, 0.125, 0.25, 0.5, and 1 mg/kg; 0.5 or 1 ml/kg) was administered 30 min prior to FR1 alcohol self-administration sessions in dependent (*n* = 10) and nondependent (*n* = 10) rats. The pretreatment time was selected based on previous work that determined peak brain oxytocin concentrations following intraperitoneal oxytocin administration [22]. The test order of intraperitoneal doses was counterbalanced using a within-subjects Latin-square design. Self-administration of alcohol and water was recorded during tests. Based on results of the FR1 test, the 0, 0.125, and 0.25 mg/kg intraperitoneal doses were then tested on a progressive ratio (PR) schedule of reinforcement, in which the number of lever presses required to obtain the next alcohol reinforcer increased progressively, as follows: 1, 1, 2, 2, 3, 3, 4, 4, 5, 5, 7, 7, 9, 9, 11, 11, 13, 13, etc. The last completed ratio (breakpoint) was used as an indication of motivation for alcohol. During the PR test, only the alcohol lever was made available. Sessions lasted 90 min or until 15 min had elapsed without a response. The pretreatment time during the PR test was the same as for the FR1 test. Again, these doses were administered in a Latin-square design, with intervening nontesting days.

### Effect of intranasal oxytocin on alcohol intake (FR1) and motivation (PR)

To reestablish a stable baseline of intake, the same rats described above were moved to different operant chambers with matching operant manipulanda and reinforcer receptacles. They were allowed eight 30-min FR1 sessions to obtain alcohol and water (S1 Fig). Next, intranasal oxytocin was administered 1 h prior to testing responding for alcohol on FR1 and PR schedules of reinforcement (described above). The pretreatment time was selected based on previous work that determined peak brain oxytocin concentrations following intranasal oxytocin administration [22]. Again, tests were conducted in Latin-square designs with intervening nontesting days. For intranasal drug delivery, general anesthesia was rapidly induced by isoflurane (5% for 2 to 5 min), and then rats were immediately administered intranasal oxytocin (0, 0.25, 0.5, and 1 mg/kg/20 μl; based on the FR1 data, the intranasal doses of 0.5 and 1 mg/kg/20 μl were selected for testing on the PR schedule) using the rat Precision Olfactory Device (rPOD; Impel NeuroPharma, Seattle, WA) and allowed to recover before being returned to their home cage.

### Effect of systemic and intranasal oxytocin on locomotion, grooming, motor coordination, and consumption of nonalcoholic palatable solutions

Based on the effects of oxytocin on alcohol drinking and the motivation for alcohol, we selected the doses of intraperitoneal (0.25 mg/kg) and intranasal (1 mg/kg) oxytocin for further behavioral testing. These doses by each route were the lowest dose by each route that significantly reduced alcohol drinking and the motivation for alcohol specifically in dependent rats. The pretreatment times were the same as used for testing on alcohol self-administration.

Behavioral tests were conducted in separate cohorts of rats to assess potential non–alcohol-specific effects of oxytocin on locomotion and grooming in an open field (dependent, *n* = 6; nondependent, *n* = 6) and motor coordination on a rotarod (dependent, *n* = 5; nondependent, *n* = 7). Two blind observers scored any instances of grooming behavior according to previously published work. A single point was given for each instance of vibration of forepaws, face washing, body grooming, body scratching, paw licking, head shaking, body shaking, and genital grooming, according to previously reported studies [40,41]. Points were totaled for each animal to yield a grooming score.

In addition to alcohol’s pharmacological effect, alcohol contains calories and has a sweet-taste component that contribute to the reinforcer efficacy of alcohol. To assess the potential role of calories and sweet taste in the ability of intraperitoneal and intranasal oxytocin to reduce alcohol consumption, we tested oxytocin on the consumption of a nonalcoholic caloric reinforcer without a sweet taste (5% maltodextrin; *n* = 7) and on a nonalcoholic sweet solution without caloric content (0.1% saccharin; *n* = 8; additional details provided in S1 Text).

### Central versus peripheral mediation of oxytocin’s effect on alcohol intake

Three separate cohorts of alcohol-dependent rats were used (central oxytocin administration: *n* = 6; peripheral oxytocin receptor agonist: *n* = 9; peripheral oxytocin receptor antagonist combined with intranasal oxytocin administration: *n* = 12). A subset of the group used for testing the peripheral oxytocin receptor antagonist combined with intranasal oxytocin administration was given 2 sessions in the absence of any treatment to reestablish a baseline and then used to test the effect of central administration of the oxytocin receptor agonist PF-06655075 (central oxytocin receptor agonist: *n* = 7). The central oxytocin and PF-06655075 administration cohorts were surgically implanted with a guide cannula to allow intracerebroventricular administration of these compounds.

The oxytocin receptor antagonist L-371,257, which does not cross the blood-brain barrier, was tested in combination with intranasal oxytocin administration to test the ability of peripheral antagonism to reverse the ability of intranasal oxytocin to reduce alcohol drinking in dependent rats. L-371,257 is a potent and competitive antagonist of the oxytocin receptor (pA2 = 8.4) with high affinity at both the oxytocin receptor (Ki = 19 nM) and vasopressin V1a receptor (Ki = 3.7 nM) [42]. The pretreatment time and dose (45 min; 5 mg/kg) were selected to be in excess of doses that provided blockade of oxytocin-induced uterine contractions for at least several hours following peripheral (intravenous or intraduodenal) administration in rats [42]. Doses much lower than this in rats (0.5 mg/kg intraperitoneal) [43] and mice (300 μg/kg intranasal) [44,45] have been demonstrated to provide behaviorally effective antagonism. Additionally, in previous studies, comparable doses of L-371,257 versus oxytocin (0.5 mg/kg versus 0.5 mg/kg intraperitoneal in rats [43]; 300 μg/kg versus 200 μg/kg intranasal in mice [45]) were administered. Here, we administered 5 times the dose of the antagonist compared to oxytocin (5 mg/kg versus 1 mg/kg) via a preferred route of peripheral absorption (intraperitoneal injection versus intranasal application [23]) to conservatively test for the ability of the peripherally acting antagonist to alter the actions of intranasal oxytocin. Rats were tested in a within-subjects Latin-square design with the following combinations of intraperitoneal and intranasal treatments: intraperitoneal vehicle with intranasal vehicle, intraperitoneal vehicle with intranasal oxytocin (1 mg/kg/20 μl), intraperitoneal L-371,257 (5 mg/kg/ml) with intranasal vehicle, and intraperitoneal L-371,257 (5 mg/kg/ml) with intranasal oxytocin (1 mg/kg/20 μl). Operant training and induction of dependence were conducted, as described above.

To test the effect of central versus peripheral oxytocin receptor agonism on dependence-induced alcohol drinking, we administered centrally or peripherally a long-acting, large molecule that does not penetrate the blood-brain barrier (PF-06655075). For central administration, 7 rats were surgically implanted with a guide cannula to allow intracerebroventricular administration of PF-06655075. These rats were previously used to test the peripheral oxytocin receptor antagonist combined with intranasal oxytocin administration (data shown in Fig 3B). The PF-06655075 dose of 30 μg was selected for intracerebroventricular administration to match the highest dose of oxytocin administered by the same route, a dose that blocked drinking in alcohol-dependent rats. For peripheral administration, the rats were administered 1 mg/kg of PF-06655075 subcutaneously. This dose was selected taking into account the high plasma protein binding of PF-06655075 (i.e., resulting in lower unbound concentrations than oxytocin) as well as its oxytocin receptor binding Ki. Subcutaneous administration of 1 mg/kg of PF-06655075 versus 1 mg/kg of oxytocin would be expected to produce comparable receptor occupancy (94.2% versus 99.7% at Cmax; see S1 Text for calculations based on data of Modi and colleagues [26]). Therefore, 1 mg/kg of subcutaneous PF-06655075 was expected to recapitulate the putative peripheral binding of oxytocin. This hypothesis was based on the observation that lower doses of intraperitoneal oxytocin were sufficient to block drinking in alcohol-dependent rats in the present study. Specifically, 0.25 mg/kg of intraperitoneal oxytocin reduced alcohol drinking in dependent rats to a similar extent as 30 μg of intracerebroventricular oxytocin. Finally, this systemic dose of 1 mg/kg PF-06655075 has been reported to reduce fear behavior potentially via anti-sympathetic action in the periphery [26].

### Electrophysiology

Dependent and nondependent rats were deeply anesthetized with isoflurane followed by rapid decapitation and immediate removal of the brain into an ice-cold high-sucrose brain slice cutting solution (sucrose 206 mM; KCl 2.5 mM; CaCl2 0.5 mM; MgCl2 7 mM; NaH2PO4 1.2 mM; NaHCO3 26 mM; glucose 5 mM; HEPES 5 mM [pH 7.4]). Coronal slices (300 to 400 μm) containing the CeA were continuously superfused (flow rate of 2 to 4 ml/min) with 95% O_2_/5% CO_2_ equilibrated aCSF of the following composition: NaCl 130 mM, KCl 3.5 mM, NaH2PO4 1.25 mM, MgSO4·7H2O 1.5 mM, CaCl2 2.0 mM, NaHCO3 24 mM, and glucose 10 mM. Recordings were performed in neurons from the medial subdivision of the CeA. Each experimental group contained neurons from a minimum of 3 rats. GABAergic activity was pharmacologically isolated with DNQX, DL-AP5, and CGP. All drugs were applied by bath superfusion.

We recorded with sharp micropipettes filled with 3M KCl and evoked GABAergic inhibitory postsynaptic potentials (eIPSPs) by stimulating locally within the medial subdivision of CeA through a bipolar electrode. Neurons were held near their resting membrane potential (−82.4 ± 0.8 mV). We performed an input–output (I/O) protocol consisting of a range of 5 current stimulations, starting at the threshold current required to elicit an eIPSP, up to the strength required to elicit the maximum subthreshold amplitude. The middle stimulus intensity was used to monitor drug-induced changes throughout the duration of the experiment. Paired-pulse ratio (PPR) was performed at the stimulus intensity giving approximately 50% of the maximal amplitude determined in the I/O protocol.

Whole-cell voltage-clamp recordings of GABAergic spontaneous inhibitory postsynaptic currents (sIPSCs) and miniature inhibitory postsynaptic currents (mIPSCs) were from visualized CeA neurons clamped at −60 mV for the duration of the recordings. Patch pipettes (3 to 6 MΩ) were filled with an internal solution composed of the following (in mM): 145 KCl, 0.5 EGTA, 2 MgCl2, 10 HEPES, 2 Na-ATP, and 0.2 Na-GTP. In all experiments, cells with a series resistance greater than 25 MΩ were excluded from analysis, and series resistance was continuously monitored during gap-free recording with a 10-mV pulse. Cells in which series resistance changed more than 25% during the course of the experiment were excluded from analysis. All measures were performed prior to (baseline) and during drug application (details in S1 Text).

### Statistical analysis

Results are presented as the mean ± standard error of the mean. The level of significance was established as *p* < 0.05. Statistical analyses were performed in Prism 6 (Graphpad Software, Inc., La Jolla, CA). Behavioral data were analyzed with one-way (Dose, Session, or Treatment) repeated-measures analysis of variance (R.M. ANOVA), two-way (Group × Dose) R.M. ANOVA, or by paired-samples *t* test. The Holms-Sidak test was used for post hoc comparisons. Electrophysiology data were analyzed with two-way ANOVA (Group × Concentration or Group × Treatment) followed by Bonferroni post hoc comparisons, one-sample *t* test, or independent-samples *t* test, as appropriate.

## Results

### Effect of intraperitoneal oxytocin on alcohol intake (FR1) and motivation (PR)

Passive exposure to alcohol vapor has been demonstrated to cause somatic signs of dependence in alcohol withdrawal as well as a dysregulation of reward and stress systems. The cardinal feature of the model is the increased consumption and motivation for alcohol exhibited by alcohol-dependent rats when allowed to perform an operant response for access to alcohol [3]. Prior to pharmacological testing, alcohol-dependent rats exposed to chronic-intermittent alcohol vapor exhibited significantly enhanced consumption of alcohol in comparison to nondependent rats that were exposed to air in their home cage throughout the study (S1 Fig).

**Fig 1:**
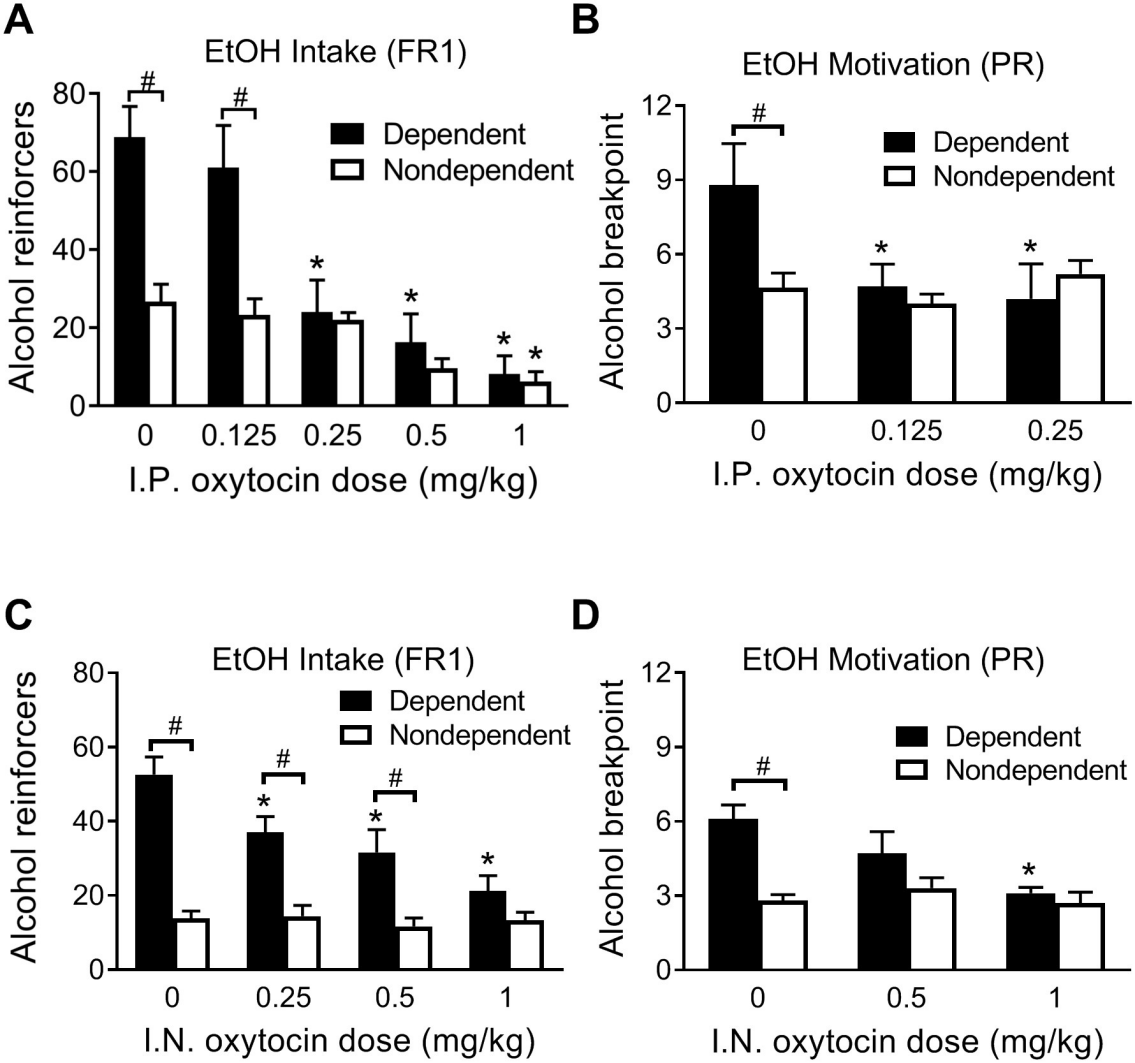
Effect of intraperitoneal and intranasal oxytocin on alcohol intake (FR1) and motivation (PR). (A) Effect of intraperitoneal oxytocin on alcohol reinforcers earned by dependent and nondependent rats in 30-min alcohol self-administration sessions. (B) Effect of intraperitoneal oxytocin on breakpoint for alcohol on a PR schedule of reinforcement. (C) Effect of intranasal oxytocin on alcohol reinforcers earned by dependent and nondependent rats in 30-min alcohol self-administration sessions. (D) Effect of intranasal oxytocin on breakpoint for alcohol on a PR schedule of reinforcement. ^#^Significant difference between dependent and nondependent rats (*p* < 0.05). *Significantly different from groups’ respective control condition (0 mg/kg; *p* < 0.05). EtOH, ethanol; FR1, fixed ratio 1; IN, intranasal; IP, intraperitoneal; PR, progressive ratio.

Oxytocin abolished the difference in alcohol drinking between dependent and nondependent rats at doses of ≥ 0.25 mg/kg (Fig 1). A 2 × 5 (Group × Dose) R.M. ANOVA yielded a significant Group × Dose interaction (*F*_4, 72_ = 7.98, *p <* 0.0001). Post hoc analyses indicated that dependent rats self-administered significantly more alcohol than the nondependent rats following 0 (*p <* 0.0001) and 0.125 mg/kg (*p <* 0.001) oxytocin doses, but this difference disappeared at higher doses. Responding was significantly lowered at the 0.25, 0.5, and 1 mg/kg doses (all *p* < 0.0001) in dependent rats compared with the vehicle condition, whereas only the highest dose of 1 mg/kg significantly lowered lever pressing for alcohol in nondependent rats (*p* < 0.05). Water intake data during all tests are presented in S2 Fig.

Oxytocin (0.125 or 0.25 mg/kg) selectively eliminated the increased motivation for alcohol in dependent rats (Fig 1B). A 2 × 3 (Group × Dose) R.M. ANOVA yielded a significant Group × Dose interaction (*F*_2, 36_ = 3.59, *p* < 0.05). The difference in PR breakpoint between groups following vehicle treatment was significant (*p* < 0.05). When treated with either 0.125 or 0.25 mg/kg doses of oxytocin, dependent rats no longer differed from nondependent rats. Oxytocin (0.125 and 0.25 mg/kg) significantly reduced PR breakpoint for alcohol in dependent rats (all *p* < 0.01), whereas responding in the nondependent group was not altered by oxytocin.

### Effect of intranasal oxytocin on alcohol intake (FR1) and motivation (PR)

Intranasal oxytocin dose-dependently decreased alcohol drinking in dependent rats without affecting drinking in nondependent rats (Fig 1C). The 2 × 4 (Group × Dose) R.M. ANOVA yielded a significant Group × Dose interaction (*F*_3, 54_ = 14.18, *p* < 0.0001). Dependent rats drank more alcohol than nondependent rats treated with saline, 0.25, and 0.5 mg/kg oxytocin (all *p* < 0.001). This difference was no longer observed following treatment with 1 mg/kg oxytocin. Alcohol drinking in dependent rats significantly decreased across the doses of oxytocin compared to the saline condition (all *p* < 0.0001), whereas drinking in the nondependent group was not significantly altered by oxytocin.

On the PR test (Fig 1D), two-way R.M. ANOVA yielded a significant Group × Dose interaction (*F*_2, 36_ = 4.27, *p* < 0.05). Following saline treatment, dependent rats had a significantly higher breakpoint for alcohol than nondependent rats (*t*_54_ = 4.50, *p* < 0.001). This difference was no longer observed following 0.5 or 1 mg/kg oxytocin administration. Oxytocin (1 mg/kg) reduced PR breakpoint in the dependent group (*p* < 0.001) without altering the behavior of nondependent rats.

### Effect of intraperitoneal and intranasal oxytocin on locomotion, grooming, rotarod motor coordination, and consumption of nonalcoholic palatable solutions

**Fig 2:**
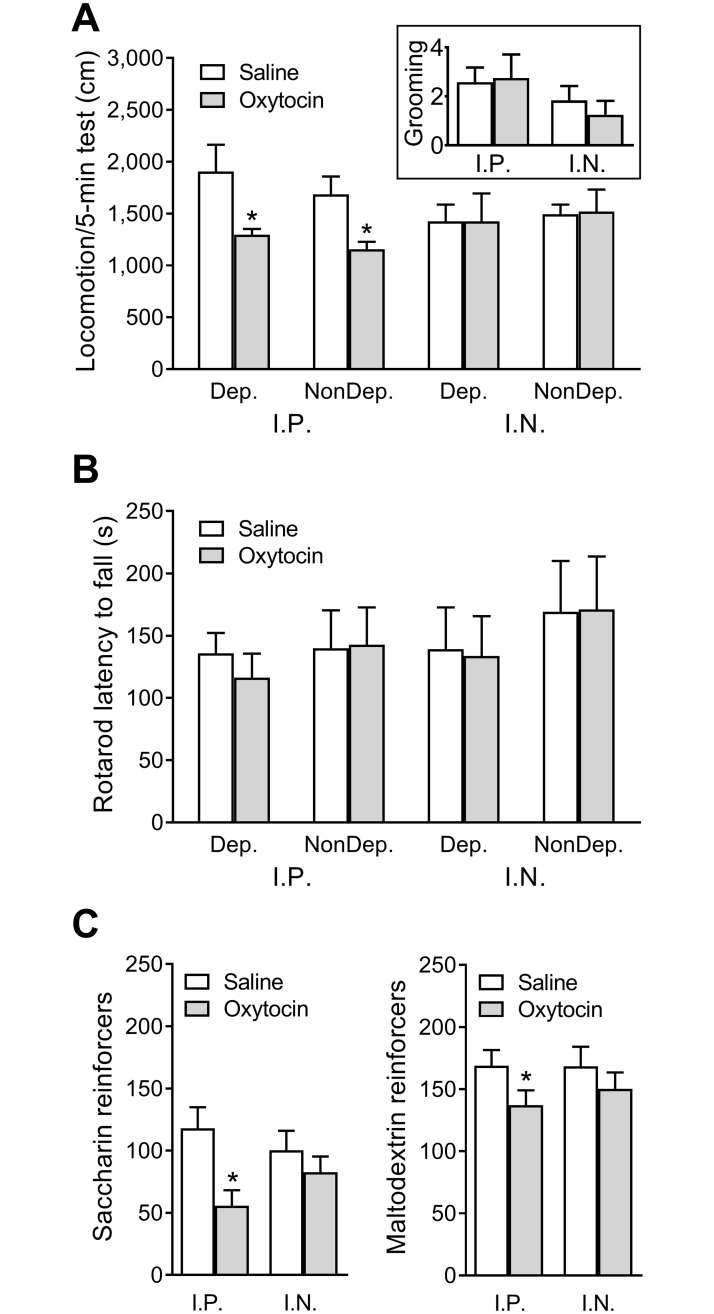
Effects of intraperitoneal and intranasal oxytocin administration on locomotion, grooming, motor coordination and consumption of nonalcoholic palatable solutions. Effect of intraperitoneal (0.25 mg/kg) and intranasal (1 mg/kg) oxytocin on (A) spontaneous locomotion measured in a 5-min test in an open field. The inset panel shows grooming behavior during the test collapsed across dependent and nondependent rats; (B) motor coordination measured in latency to fall in an accelerating rotarod test; (C) consumption of palatable nonalcoholic solutions assessed in rats trained on an FR1 schedule to respond for access to sweet/noncaloric 0.1% saccharin or caloric/nonsweet 5% maltodextrin. *Significantly different from saline (*p* < 0.05). Dep, dependent; FR1, fixed-ratio 1; IN, intranasal; IP, intraperitoneal; NonDep, nondependent.

Dependent and nondependent rats were not significantly different in their spontaneous locomotion or grooming behavior. Intraperitoneal oxytocin (0.25 mg/kg) significantly decreased locomotion in both dependent and nondependent rats (Fig 2A; Dose: *F*_1, 10_ = 14.63, *p* < 0.01; post hoc tests all *p* < 0.05), whereas intranasal oxytocin (1 mg/kg) had no effect in either group. Intraperitoneal and intranasal oxytocin treatments did not affect grooming behavior (which remained minimal throughout testing; Fig 2A inset). Dependent and nondependent rats did not significantly differ in their rotarod performance, and this performance was not significantly altered by intraperitoneal or intranasal oxytocin treatments (Fig 2B). Intraperitoneal oxytocin reduced saccharin (*t*_6_ = 2.80, *p* < 0.05) and maltodextrin (*t*_6_ = 3.20, *p* < 0.05) consumption, whereas intranasal oxytocin had no effect (Fig 2C).

### Central versus peripheral effect of oxytocin on alcohol intake

**Fig 3:**
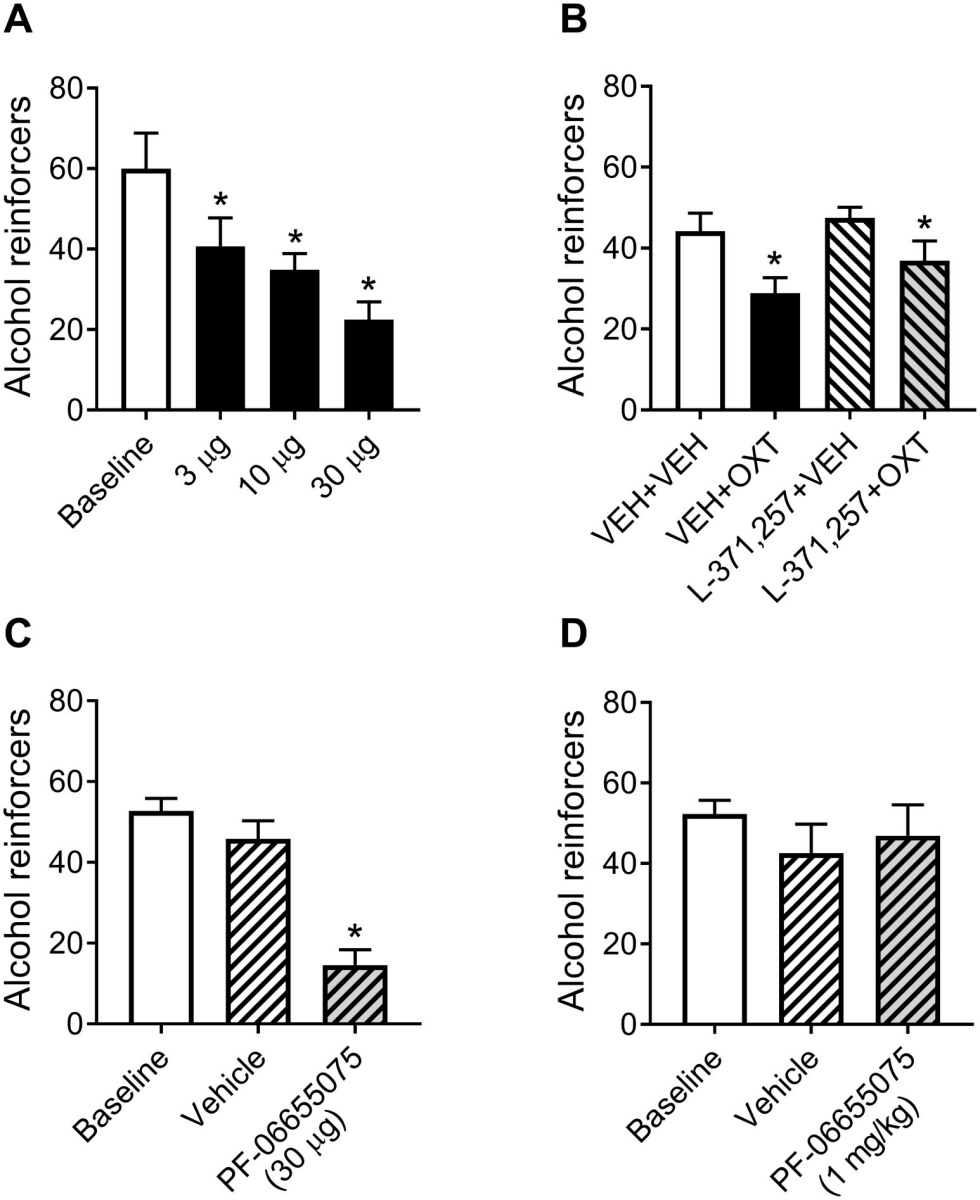
Central versus peripheral mediation of OXT’s effect on alcohol intake. (A) Effect of intracerebroventricular infusion of OXT on alcohol reinforcers earned by dependent rats in 30-min alcohol self-administration sessions. (B) Effect of peripheral administration (intraperitoneal) of the OXT receptor antagonist L-371,257 that does not cross the blood-brain barrier on the ability of intranasal OXT to reduce alcohol reinforcers earned by dependent rats in 30-min alcohol self-administration sessions. (C) Effect of central (intracerebroventricular) administration of the OXT receptor agonist (PF-06655075, a long-acting, large molecule that does not cross the blood-brain barrier), on reinforcers earned by dependent rats in 30-min alcohol self-administration sessions. (D) Effect of systemic (subcutaneous) administration of PF-06655075 on reinforcers earned by dependent rats in 30-min alcohol self-administration sessions. *Significantly different from baseline or VEH condition (both in the case of panel C) (*p* < 0.05). OXT, oxytocin; VEH, vehicle.

Baseline sessions conducted between intracerebroventricular oxytocin test sessions did not significantly differ, so they were combined for analysis (Fig 3A). All doses of intracerebroventricular oxytocin reduced alcohol consumption in dependent rats (Dose: *F*_3, 15_ = 10.25, *p* < 0.001; post hoc tests: all *p* < 0.05).

Intranasal oxytocin treatment significantly reduced drinking in dependent rats whether the rats were pretreated with either vehicle (VEH + VEH versus VEH + OXT) or with the peripherally restricted oxytocin receptor antagonist L-371,257 (L-371,257 + VEH versus L-371,257 + OXT; Treatment: *F*_3, 33_ = 10.73, *p* < 0.0001; post hoc tests: all *p* < 0.05). L-371,257 did not significantly alter drinking by itself (VEH + VEH versus L-371,257 + VEH) nor did it reverse the ability of intranasal oxytocin to reduce alcohol drinking in dependent rats (VEH + OXT versus L-371,257 + OXT; Fig 3B).

Baseline sessions conducted between test sessions with intracerebroventricular administration of PF-06655075 did not significantly differ and thus were combined for analysis (Fig 3C). Intracerebroventricular administration of PF-06655075, which does not cross the blood-brain barrier and thus was expected to not diffuse to the periphery, significantly reduced responding for alcohol relative to baseline responding and relative to vehicle (Treatment: *F*_2, 12_ = 42.25, *p* < 0.0001; post hoc tests: all *p* < 0.0001). Vehicle administration did not significantly alter responding relative to baseline (Fig 3C). Systemic administration of PF-06655075 (expected to not reach the brain) did not significantly alter responding for alcohol relative to baseline responding or the vehicle condition nor did the vehicle itself significantly alter responding relative to baseline (Fig 3D).

### Oxytocin decreases evoked CeA GABAergic signaling in nondependent but not dependent rats

We recorded from neurons in the medial subdivision of the CeA, using sharp intracellular whole cell configuration for locally evoked inhibitory GABAergic eIPSP and whole-cell patch-clamp configuration for GABAergic postsynaptic currents. We found no difference in input resistance of neurons from nondependent (154.4 ± 11.4 MΩ) and dependent (159.7 ± 7.6 MΩ) rats and no difference in spike frequency (S3 Fig) between groups. Baseline GABAergic eIPSP amplitudes stimulated locally within the medial subdivision of CeA were not different between nondependent (9.7 ± 0.7 mV) and dependent (9.9 ± 0.7 mV) animals (S4 Fig). No differences were observed in PPR of eIPSPs at 100 ms interstimulus interval in nondependent (1.06 ± 0.08) and dependent (1.01 ± 0.09) neurons.

**Fig 4:**
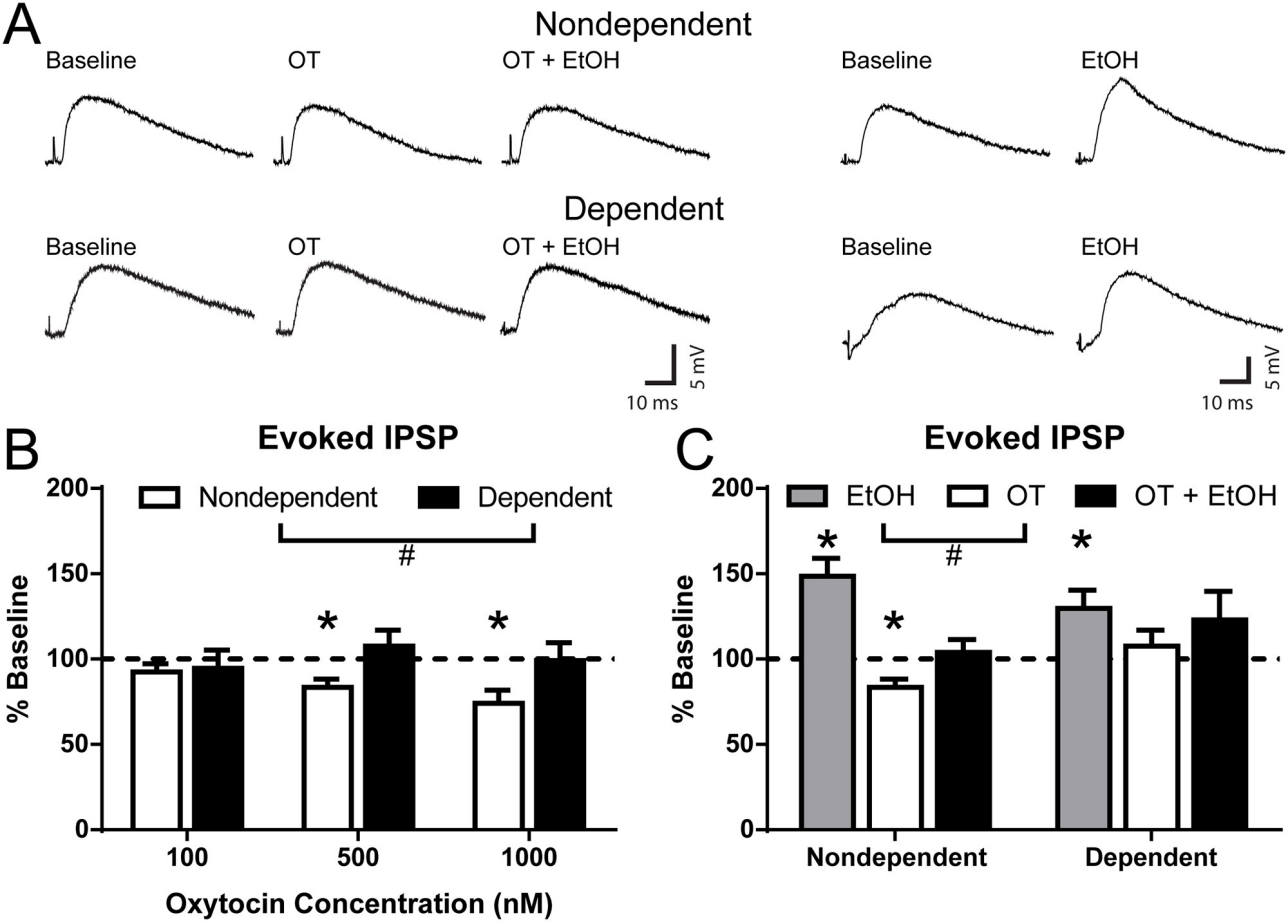
Oxytocin decreases locally evoked CeA GABAergic signaling and blunts alcohol effects. (A) Representative eIPSPs recorded from CeA neurons of nondependent (upper) and dependent (lower) rats before (baseline) and during acute application of oxytocin (500 nM), oxytocin with alcohol (44 mM), and alcohol alone. (B) Effect of 3 concentrations of oxytocin (100, 500, and 1,000 nM) on eIPSP amplitudes in CeA neurons from alcohol-nondependent and -dependent rats. Higher concentrations of oxytocin (500 and 1,000 nM) decreased eIPSP amplitudes in nondependent but not in dependent rats. (C) Oxytocin blunted the alcohol-induced enhancement of eIPSP in neurons from both alcohol-nondependent and alcohol-dependent rats. *Significant effect of drug compared to baseline (*p* < 0.05). ^#^Significant difference between groups (*p* < 0.05). CeA, central nucleus of the amygdala; eIPSP, evoked inhibitory postsynaptic potential; EtOH, ethanol; OT, oxytocin.

In CeA neurons of nondependent rats, 100 nM oxytocin (10 to 15 min) did not alter eIPSP amplitudes, whereas 500 nM and 1,000 nM significantly decreased amplitudes to 83.4% ± 4.9% (*t*_10_ = 3.40, *p* < 0.01) and 74.1% ± 7.7% (*t*_4_ = 3.37, *p* < 0.05) of baseline, respectively (Fig 4B). Notably, in dependent rats, oxytocin at all 3 concentrations did not affect eIPSPs. A two-way ANOVA (Group × Concentration) yielded a significant Group effect (*F*_1,44_ = 4.55, *p* < 0.05). Oxytocin did not alter input resistance of nondependent (baseline: 144.3 ± 13.8 MΩ, oxytocin: 148.3 ± 13.7 MΩ) or dependent (baseline: 148.2 ± 10.6 MΩ, oxytocin: 141.8 ± 12.9 MΩ) CeA neurons, suggesting that oxytocin effects on eIPSP amplitudes were not due to excitability changes but due to decreased GABAergic transmission. Oxytocin (500 nM) significantly increased the PPR of eIPSPs (baseline PPR: 0.98 ± 0.11; oxytocin PPR: 1.35 ± 0.14; t_10_ = 2.56, *p* < 0.05) in nondependent rats, suggesting a possible decrease in the presynaptic release of GABA (as changes in PPR are inversely related to changes in release) [46]. Oxytocin did not alter PPR in dependent rats (baseline PPR: 1.17 ± 0.22, oxytocin PPR: 1.03 ± 0.07).

### Alcohol increases evoked CeA GABAergic transmission

Acute alcohol (44 mM; maximal dose [47]) application significantly increased eIPSP amplitudes in CeA of both nondependent (to 140.8% ± 8.5% of baseline; *t*_7_ = 4.79, *p* < 0.01) and dependent rats (to 133.9% ± 13.4% of baseline; *t*_6_ = 2.53, *p* < 0.05; Fig 4C), indicating a lack of tolerance for the acute effects of alcohol on stimulated GABAergic signaling, as previously reported [47]. Additionally, alcohol did not alter input resistance of nondependent (baseline: 169.0 ± 19.2 MΩ, alcohol: 167.6 ± 15.8 MΩ) or dependent (baseline: 167.6 ± 10.4 MΩ, alcohol: 161.4 ± 11.4 MΩ) neurons. Because oxytocin also binds to vasopressin receptors and there is a CeA subpopulation of neurons sensitive to vasopressin binding (specifically, via the V1a but not V1b vasopressin receptor), we always preapplied the selective vasopressin receptor 1A antagonist TMA (500 nM) to isolate specific oxytocin receptor-mediated actions [34,48]. TMA had no effect on eIPSP amplitudes (nondependent: 8.51 ± 1.08 mV and dependent: 9.68 ± 1.31 mV without TMA) and did not affect the magnitude of the alcohol-induced increase of eIPSPs in both nondependent (to 148.6% ± 10.5% of baseline; *t*_5_ = 4.64, *p* < 0.01) and dependent rats (129.8% ± 10.6% of baseline; *t*_6_ = 2.80, *p* < 0.05; S5 Fig).

To determine potential interactions between oxytocin and acute alcohol, in most of the neurons that received 500 nM oxytocin, we applied alcohol in the presence of oxytocin. In this subset of neurons (7 of 9) from nondependent slices, coapplication of alcohol only induced a moderate, nonsignificant increase of eIPSP amplitudes (oxytocin: 83.4% ± 4.9%; oxytocin + alcohol: 101.7% ± 8.3% of baseline; Fig 4C). In the subset of neurons (12 of 15) from dependent slices, alcohol did not further increase eIPSPs (oxytocin: 107.6% ± 9.3%; oxytocin + alcohol: 123.0% ± 16.8% of baseline; Fig 4C), suggesting a blockade of oxytocin-induced decreased GABA transmission in dependent rats. A two-way ANOVA (Group × Treatment) yielded a significant main effect of Treatment (*F*_2,53_ = 6.33, *p* < 0.01), and a Bonferroni post hoc test indicated a significant difference between the effects of alcohol and oxytocin (*t*_53_ = 3.35, *p* < 0.01). Similarly, oxytocin at 100 and 1,000 nM blunted the alcohol-induced facilitation of eIPSPs in both nondependent (84.2% ± 11.0% and 82.1% ± 13.2% of baseline, respectively) and dependent slices (124.0% ± 18.6% and 125.5% ± 15.3% of baseline, respectively). Overall, oxytocin blunted the alcohol-induced increase of GABA transmission (see Fig 4C).

### Oxytocin decreases CeA GABA_A_ receptor function

To further investigate the pre- versus postsynaptic action of oxytocin on GABA signaling, we performed whole-cell patch-clamp recordings of sIPSCs and mIPSCs in CeA neurons. Generally, changes in IPSC frequency reflect altered transmitter release, and changes in amplitude or kinetics reflect alterations in postsynaptic GABA_A_ receptor sensitivity. However, altered amplitude may also reflect a mix of pre- and postsynaptic effects [49,50].

**Fig 5:**
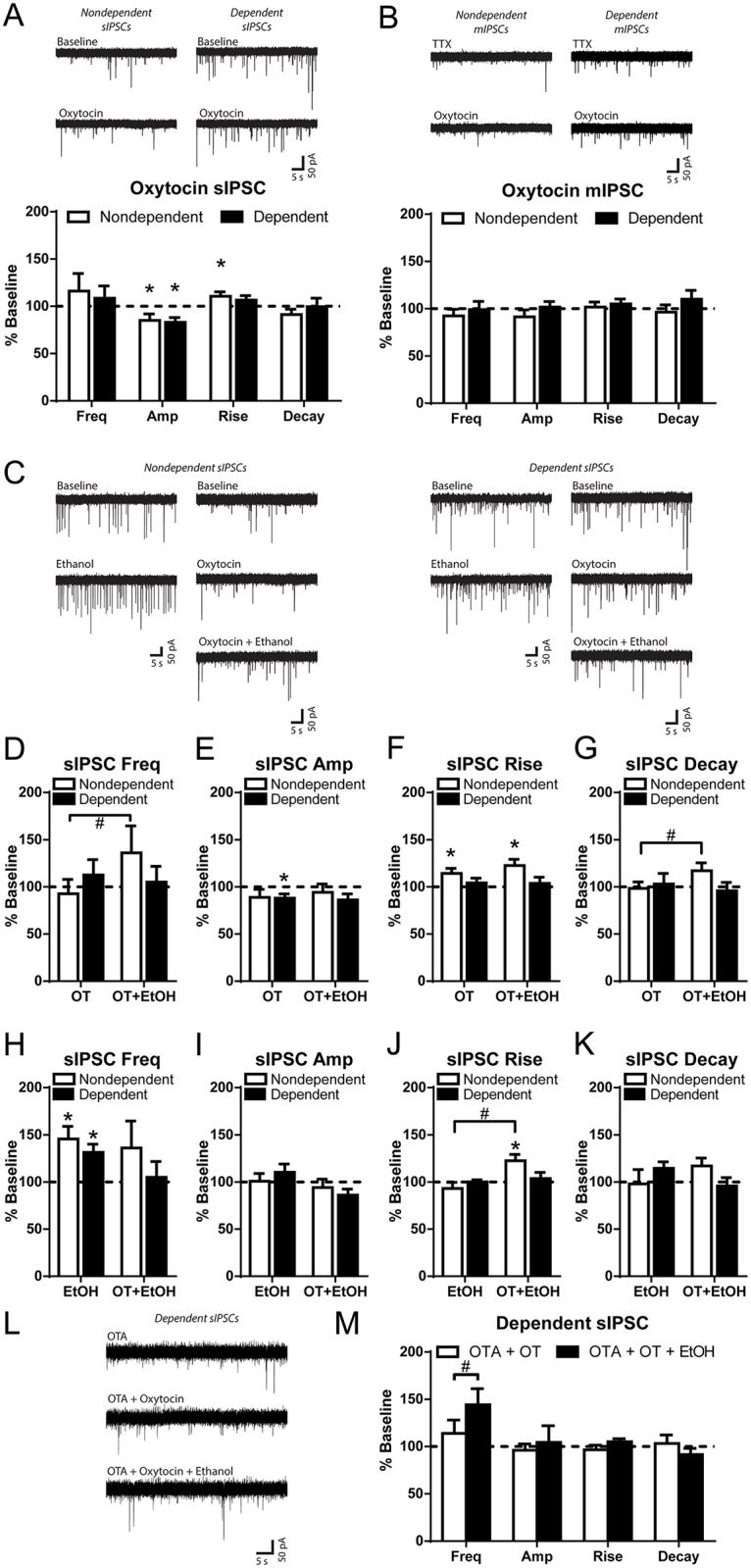
Oxytocin decreases postsynaptic CeA spontaneous GABAergic transmission and blocks presynaptic alcohol effects. (A, Top) Representative GABA_A_-mediated sIPSCs of CeA neurons from nondependent and dependent rats prior to (baseline) and during application of oxytocin (500 nM). (A, Bottom) Oxytocin decreased sIPSC amplitude from baseline in CeA neurons from both nondependent and dependent rats and caused a small increase from baseline in sIPSC rise time in nondependent rats. However, there were no significant differences between neurons from nondependent and dependent rats across these measures. (B, Top) Representative GABA_A_-mediated mIPSCs of CeA neurons from nondependent and dependent rats with TTX and during application of oxytocin (500 nM). (B, Bottom) Oxytocin had no effect on mIPSC frequency, amplitude, or kinetics in CeA neurons from both nondependent and dependent rats. (C) Representative sIPSCs of CeA neurons from nondependent (Left) and dependent (Right) rats before and during application of alcohol (44 mM), oxytocin (500 nM), and oxytocin with alcohol. (D) In the neurons that received oxytocin and oxytocin with alcohol, oxytocin had no significant effect on sIPSC frequency from baseline in either nondependent or dependent rats. However, alcohol with oxytocin significantly increased sIPSC frequency from oxytocin alone only in nondependent rats. (E) In these same neurons, oxytocin decreased amplitude of sIPSCs from baseline in dependent rats but with no significant effects of drug or alcohol history. (F) Oxytocin and coapplication of oxytocin with alcohol increased rise time from baseline in neurons from nondependent rats only, with no significant effects of drug or alcohol history. (G) Alcohol with oxytocin increased sIPSC decay times from oxytocin alone only in nondependent rats. (H) Alcohol alone increased frequency of sIPSCs from baseline in both nondependent and dependent rats but with no significant effects of drug or alcohol history when compared to alcohol with oxytocin. Alcohol had no effect on amplitude (I), rise times (J), or decay times (K) in nondependent or dependent rats, but the alcohol with oxytocin increase in rise times in nondependent rats was significantly different from the effect of alcohol alone (J). (L) Representative sIPSCs of CeA neurons from dependent rats with the oxytocin receptor antagonist OTA (100 nM), during oxytocin (500 nM), and during subsequent coapplication of oxytocin with 44 mM alcohol. (M) OTA blocked oxytocin’s effects on sIPSC amplitude and subsequent alcohol application increased sIPSC frequency. *Significantly different from baseline or control condition (*p* < 0.05). ^#^Significant difference between groups (*p* < 0.05). Amp, amplitude; CeA, central nucleus of the amygdala; EtOH, ethanol; Freq, frequency; mIPSC, miniature inhibitory postsynaptic current; OT, oxytocin; OTA, desGly-NH2-d(CH2)5[D-Tyr2,Thr4]OVT; sIPSC, spontaneous inhibitory postsynaptic current; TTX, tetrodotoxin.

We first investigated sIPSCs, finding no significant differences between nondependent and dependent rats in sIPSC frequency (1.1 ± 0.2 and 0.8 ± 0.1 Hz, respectively), amplitude (82.0 ± 8.4 and 66.6 ± 5.8 pA, respectively), rise time (2.6 ± 0.1 and 2.8 ± 0.1 ms, respectively), or decay time (8.0 ± 0.8 and 9.4 ± 1.0 ms, respectively). We next used the vasopressin receptor 1A antagonist TMA and found no effects of TMA alone in either nondependent or dependent rats (S6 Fig), as observed for evoked GABAergic responses. In CeA neurons of nondependent rats, application of 500 nM oxytocin significantly decreased the amplitude of sIPSCs to 85.2% ± 6.7% of baseline (*t*_11_ = 2.22, *p* < 0.05) and increased rise times to 110.5% ± 4.7% of baseline (*t*_11_ = 2.24, *p* < 0.05) with no changes in frequency or decay times (Fig 5A), indicating mainly postsynaptic actions of oxytocin to decrease GABA_A_ receptor function. In dependent rats (Fig 5A), oxytocin also significantly decreased sIPSC amplitude to 83.1% ± 5.0% of baseline (*t*_8_ = 3.37, *p* < 0.01), with no changes in sIPSC frequency or kinetics.

We next investigated mIPSCs in the presence of the sodium channel blocker TTX to isolate action potential independent currents. Generally, mIPSC analysis reveals specific effects of drugs on vesicular release of GABA, which results from exocytosis of neurotransmitter containing vesicles in a manner independent of action-potential–induced release mechanisms. In addition, sIPSCs and mIPSCs likely result from distinct presynaptic neurotransmitter release mechanisms, e.g., vesicle fusion machinery, spatial segregation of the vesicles and/or vesicle populations, and synaptic vesicle pool dynamics [51]. Similar to sIPSCs, we did not find any baseline differences between nondependent and dependent rats in mIPSC frequency (0.8 ± 0.1 and 0.9 ± 0.3 Hz, respectively), amplitude (53.2 ± 4.0 and 60.7 ± 12.3 pA, respectively), rise time (2.5 ± 0.1 and 2.5 ± 0.1 ms, respectively), or decay time (5.5 ± 0.6 and 6.2 ± 0.8 ms, respectively). Oxytocin did not alter mIPSC frequency, amplitude, or kinetics for neurons from nondependent or dependent rats (Fig 5B), suggesting no effect of oxytocin on this form of GABA release in the medial subdivision of CeA.

### Oxytocin blocks alcohol’s effects on GABA release in dependent rats

We next examined the interaction of oxytocin and acute alcohol on CeA sIPSCs in nondependent and dependent rats. In 8 of the 12 CeA neurons from nondependent rats that received 500 nM oxytocin, and 7 of the 9 neurons from dependent rats, we coapplied alcohol (44 mM) and compared effects between groups on sIPSC measures (Fig 5D–5G). A two-way ANOVA (Group × Treatment) yielded a significant effect of Treatment (*F*_1,13_ = 4.87, *p* < 0.05) and a Group × Treatment interaction (*F*_1,13_ = 9.84, *p* < 0.01) for sIPSC frequency (Fig 5D). The Bonferroni post hoc comparisons indicated that alcohol with oxytocin significantly increased sIPSC frequency compared with oxytocin alone in nondependent rats (*t*_13_ = 3.91, *p* < 0.01). Therefore, oxytocin blocked the alcohol-induced increase in GABA release only in CeA neurons of alcohol-dependent rats. Additionally, a two-way ANOVA (Group × Treatment) yielded a significant Group × Treatment interaction (*F*_1,13_ = 20.88, *p* < 0.001) for sIPSC decay time (Fig 5G). The Bonferroni post hoc comparisons indicated that alcohol with oxytocin significantly increased decay times compared with oxytocin alone in nondependent rats (*t*_13_ = 4.86, *p* < 0.001).

As previously shown [52], we found that in nondependent and dependent rats, acute alcohol alone significantly increased sIPSC frequency to 145.5% ± 13.4% of baseline (*t*_5_ = 3.40, *p* < 0.05) and 131.4% ± 8.6% of baseline (*t*_9_ = 3.65, *p* < 0.01), respectively (Fig 5H), with no changes in sIPSC amplitude or kinetics, suggesting increased action-potential–dependent GABA release. We compared the effects of alcohol alone with the effects of alcohol and oxytocin using two-way ANOVAs (Group × Treatment) for each sIPSC measure (Fig 5H–5K). We found a significant main effect of Treatment (*F*_1,27_ = 9.00, *p* < 0.01) and a significant Group × Treatment interaction (*F*_1,27_ = 5.61, *p* < 0.05) for sIPSC rise times (Fig 5J). The Bonferroni post hoc comparisons indicated a significant difference in the effects of alcohol with oxytocin versus alcohol alone for nondependent neurons.

Finally, we found that the selective oxytocin receptor antagonist desGly-NH2-d(CH2)5[D-Tyr2,Thr4]OVT alone did not alter sIPSCs (S7 Fig), suggesting no basal activity of these receptors on spontaneous GABAergic transmission in dependent rats. Notably, in neurons from dependent rats, oxytocin in the presence of OTA had no effect on sIPSCs when compared to baseline (Fig 5L and 5M). In a subset of these neurons (8 out of 11), we applied alcohol in the presence of both OTA and oxytocin, resulting in a significant increase in sIPSC frequency (*t*_7_ = 3.01, *p* < 0.05; Fig 5M). We found a similar effect of OTA to rescue the alcohol effect on eIPSPs from nondependent neurons (*t*_7_ = 2.70, *p* < 0.05; S8 Fig). These results confirm that oxytocin receptor antagonism blocked postsynaptic effects of oxytocin and restored the acute alcohol-induced facilitation of GABA release in the CeA of alcohol-dependent rats.

## Discussion

Oxytocin delivered via intraperitoneal, intranasal, and intracerebroventricular routes blocked the enhanced motivation for alcohol drinking that developed in alcohol-dependent rats. Intraperitoneal and intranasal oxytocin at certain doses blocked the increased alcohol consumption and motivation for alcohol in dependent rats, without impacting these behaviors in nondependent rats. Intranasal oxytocin did not disrupt spontaneous locomotion, grooming behavior, motor coordination, or consumption of sweet or caloric palatable solutions. Central administration of oxytocin produced a dose-dependent reduction in alcohol drinking in dependent rats, and this effect was replicated by central administration of an oxytocin receptor agonist (PF-06655075) that does not cross the blood-brain barrier and therefore was expected to not diffuse to the periphery. However, peripheral administration of PF-06655075 (expected to not reach the brain) had no effect. Additionally, peripheral administration of an oxytocin receptor antagonist that does not cross the blood-brain barrier did not reverse the ability of intranasal oxytocin to reduce alcohol drinking in dependent rats. Together, the data suggest a central mechanism for oxytocin’s actions on alcohol drinking. *Ex vivo* electrophysiology recordings indicated that oxytocin inhibits spontaneous action-potential–dependent GABAergic transmission in CeA slices from both dependent and nondependent rats. However, oxytocin’s ability to dose-dependently reduce evoked network GABAergic activity was absent in slices from dependent rats. Furthermore, oxytocin blunted acute alcohol-induced facilitation of evoked GABAergic responses in both nondependent and dependent rats but blocked alcohol-induced facilitation of spontaneous action-potential–dependent GABA release only in CeA slices from alcohol-dependent rats, suggesting differential GABA network effects in nondependent and dependent rats produced by oxytocin.

Systemic oxytocin blocked the enhanced motivation for alcohol observed in dependent rats, indexed by escalated alcohol drinking and increased breakpoint in a PR test, at doses that did not change the behavior of nondependent rats. These findings are consistent with previous reports that oxytocin can decrease alcohol drinking in nondependent mice and rats [20,21] and that oxytocin is particularly effective in decreasing cue-induced reinstatement of alcohol seeking in postdependent rats compared with nondependent rats [17]. The present findings are unique in several ways from previous work in rats [17]. They demonstrate the following: (1) Oxytocin can reduce alcohol drinking and the enhanced motivation to “work” for alcohol in currently alcohol-dependent rats. This difference is critical, because it provides a novel indication for oxytocin’s potential therapeutic value. Alcohol drinking during acute (i.e., currently alcohol-dependent rats) and protracted abstinence (i.e., alcohol seeking in rats with a history of alcohol dependence) are proposed to model distinct phases and aspects of alcohol use disorder, with distinct underlying neurocircuitry [2]. The results of the present study indicate that oxytocin may have the potential to reduce heavy drinking in moderate to severe alcohol use disorder. (2) Oxytocin’s anti-drinking effects in rats can be achieved by intranasal administration, using a device designed to allow noninvasive drug delivery across the blood-brain barrier [53]. (3) Intranasal administration reduced alcohol consumption and motivation for alcohol in dependent rats without causing nonspecific locomotor, grooming, motor coordination, and consumption of nonalcoholic sweet or caloric palatable solutions, suggesting that oxytocin’s effects on alcohol drinking are specific to the pharmacological effects of alcohol. (4) The present study provides evidence that peripheral receptors did not make a major contribution to the effect of intranasal oxytocin. More specifically, intracerebroventricular administration of a large molecule (PF-06655075) that does not cross the blood-brain barrier or intracerebroventricular administration of oxytocin significantly reduced alcohol drinking in dependent rats. However, PF-06655075 administered systemically at a dose matching the highest tested dose of systemic oxytocin (4 times the dose sufficient to block drinking in alcohol-dependent rats) did not have an effect. Therefore, oxytocin’s effect of reducing alcohol drinking in the present model of alcohol dependence is likely centrally mediated. Further supporting this hypothesis, application of the nonbrain penetrant antagonist L-371,257 did not reverse the ability of intranasal oxytocin to reduce alcohol drinking in alcohol-dependent rats, suggesting that intranasal administration of oxytocin reduces alcohol drinking in dependent rats independent of oxytocin receptor binding in the periphery.

Despite the precise mechanism remaining unknown, there is accumulating evidence that peripherally applied oxytocin can cross the blood-brain barrier in adult rodents. As suggested by others [22,23], there is likely a mechanism for direct transport of intranasally applied oxytocin across the blood-brain barrier in adult rodents. Tanaka and colleagues applied oxytocin by intravenous, intraperitoneal, and intranasal routes in rats to determine the peripheral and central levels of oxytocin resulting from each route of administration [23]. The authors used the intraperitoneal administration data to determine the amount of centrally detected oxytocin that can be expected to result from peripherally circulating levels. They used this information to account for centrally detected oxytocin following intranasal administration that may be the result of oxytocin transferring first from the nasal epithelium into the peripheral circulation, then into the brain. It was concluded that, under the conditions of intranasal administration, >95% of the oxytocin detected in the brain is expected to result from direct transport across the blood-brain barrier [22,23]. Indeed, oxytocin detected in the brain was higher following intranasal administration compared with intravenous or intraperitoneal administration, despite intranasal administration resulting in far lower oxytocin plasma concentrations. Furthermore, oxytocin concentrations were especially high in the olfactory bulb after intranasal administration, suggesting a direct route of entry [23]. Bustion and colleagues demonstrated that radio-labeled oxytocin administered in mice intranasally was detected throughout the brain, i.e., dissociating exogenously applied deuterated oxytocin from endogenous oxytocin, thus confirming direct entry [24]. Again, particularly elevated levels were detected in the olfactory bulb, suggesting a point of entry at the nasal epithelium.

Although intraperitoneal oxytocin administration decreased locomotion in the open field and the consumption of a nonalcoholic, noncaloric sweet solution and a nonalcoholic, unsweetened caloric solution, intranasal administration did not. Neither intraperitoneal nor intranasal oxytocin administration altered grooming behavior in the open field or performance in the rotarod task, indicating that the intraperitoneal effect on locomotion is likely not a result of altered motor coordination. A possible explanation for nonspecific effects of intraperitoneal oxytocin administration is that that oxytocin may have “side effects” that result from binding at peripheral sites (e.g., gut, heart, vascular system, and/or vagus nerve) that disrupt behavior in general and that were induced following intraperitoneal oxytocin administration in the present study. Congruent with this account is the observation that intraperitoneal oxytocin suppressed water consumption during tests for oxytocin’s effect on alcohol consumption. In contrast, oxytocin treatment did not alter water consumption when administered intranasally, intracerebroventricularly, or intranasally in combination with L-371,257, suggesting that peripheral “side effects” were avoided during these tests (S2 Fig). Intraperitoneal administration of oxytocin, unlike the peripherally restricted agonist PF-06655075, was able to block alcohol drinking in dependent rats, similarly to intranasal oxytocin administration. Although the transfer of peripherally circulating oxytocin to the central compartment is expected to be very limited, a rapid, dramatic spike in plasma-oxytocin concentrations has been noted following intraperitoneal administration that may allow pharmacologically relevant central concentrations of oxytocin to be achieved, especially following application of supraphysiological oxytocin doses, as used in the present study [18,20,22,23,54]. A recent study found deuterated oxytocin administered intravenously was detected centrally in rhesus macaques (i.e., dissociating exogenous labeled oxytocin from endogenous oxytocin, confirming direct transfer [55]). Although intraperitoneal administration of oxytocin induced some side effects in the present study, the action of intraperitoneal oxytocin that causes reduction of alcohol drinking in dependent rats is likely the same central mechanism as engaged following intranasal administration. Nevertheless, the present data suggest that administration by intranasal compared with intraperitoneal route may result in more favorable pharmacokinetics for achieving central over peripheral oxytocin exposure [22,23].

To further test the hypothesis of central oxytocin action, we demonstrated that intracerebroventricular infusion of oxytocin and PF-06655075 (expected to not diffuse to the periphery) reduced alcohol drinking in dependent rats. Systemic administration of PF-06655075 (expected to not to cross the blood-brain barrier) did not affect alcohol intake in dependent rats. Finally, the peripherally restricted oxytocin receptor antagonist L-371,257 did not alter the effects of intranasal oxytocin in reducing alcohol drinking in dependent rats. Together, these data suggest that peripheral receptors make a minimal contribution to intranasal oxytocin’s effects on alcohol drinking.

Next, we examined the effects of oxytocin on GABAergic transmission in the CeA, a key brain region of dysregulation in alcohol dependence [3,56–59]. To gain mechanistic insight into the contribution of the oxytocin system on CeA GABAergic transmission in the context of acute and chronic alcohol, here, we examined both spontaneous action-potential–dependent and evoked GABAergic transmission wherein the network activity is intact, as well as action-potential–independent transmission. We found that oxytocin decreases GABA signaling in the CeA of both dependent and nondependent rats, but its effects varied between different modes of GABAergic transmission. In alcohol-nondependent rats, oxytocin decreased evoked GABA responses by decreasing evoked GABA release, an effect that is no longer observed in dependent rats, suggesting neuroadaptations of the CeA oxytocin system in dependence. In contrast, oxytocin decreased GABA_A_ receptor function in both nondependent and dependent rats. Similar to previous studies in the medial subdivision of CeA [48], oxytocin did not affect action-potential–independent GABAergic transmission in either group. Evoked GABA responses are generated by delivering a controlled electrical stimulation locally within the CeA, whereas spontaneous events reflect inhibitory signaling across the broader CeA synaptic network. Miniature currents result from spontaneous presynaptic vesicle fusion independent of sodium entry and more precisely determine pre- and postsynaptic drug effects occurring at the terminals. In addition, spontaneous and evoked forms of GABA transmission may represent distinct forms of GABA transmission, resulting from distinct presynaptic neurotransmitter release mechanisms (e.g., vesicle fusion machinery, spatial segregation of the vesicles and/or vesicle populations, synaptic vesicle pool dynamics) [51]. Of note, oxytocin had no effect on mIPSCs or cellular excitability, therefore its effects on sIPSC amplitude and rise time, as well as in eIPSP amplitude and PPR, suggest synaptic effects rather than cell excitability. These synaptic effects likely occur upstream within the local intact GABAergic network that is shared by spontaneous and evoked forms of synaptic transmission, whereas miniature effects are more localized to the specific terminals in the medial subdivision of the CeA where oxytocin receptors may not be present [48]. These considerations are critical and need to be taken into account when comparing with oxytocin electrophysiological effects reported on by others in the lateral subdivision of the CeA [48,60,61]. In contrast, these lateral CeA studies reported effects of oxytocin in increasing excitability of lateral CeA GABAergic neurons that project to the medial CeA, resulting in an increase in sIPSC frequency and decreased excitability in the medial CeA [48]. Although the lack of oxytocin effect on mIPSCs in the medial CeA is consistent with our results, we observed that oxytocin decreased GABAergic transmission in the medial CeA without changing cellular excitability. There are two likely reasons for these discrepancies. The first is the use of the native peptide oxytocin in our studies as opposed to the selective peptide oxytocin-receptor agonist (Thr⁴,Gly⁷)-Oxytocin (TGOT) [34]. Although we pharmacologically block the predominant vasopressin receptor in the CeA, native versus selective peptide agonists for the oxytocin receptor may exhibit functional selectivity at the oxytocin receptor or off-target effects. The second possibility is the heterogeneity and interconnectivity of GABAergic neurons in the CeA. The medial CeA contains GABAergic neurons that project out of the CeA as well as synapse locally. In one previous study [48] of the TGOT responsive cells, only about half of the TGOT inhibited neurons were excited by vasopressin. Additionally, Viviani and colleagues reported that projection-specific populations of medial CeA GABAergic neurons were unresponsive to TGOT [60]. Therefore, it is possible that the majority of the neurons from which we recorded were from a subpopulation of GABA neurons that did not receive direct innervation of oxytocin-excited lateral CeA GABAergic neurons. Additionally, from our evoked experiments, the electrical stimulation likely excited neurons in the lateral and medial CeA, and our responses were a composite of these effects. Therefore, future experiments will determine specific cell types to understand the distinct oxytocin effects on the different components of the synaptic network.

Understanding the physiological actions of oxytocin in the CeA may benefit by comparison to the pro-stress, pro-drinking effects of corticotropin-releasing factor (CRF) and the anti-stress, anti-drinking effects of neuropeptide Y (NPY). The pro-stress neuropeptide CRF is elevated in the CeA, likely mediated by glucocorticoid receptors [62,63] in alcohol dependence, and infusion of CRF_1_ or glucocorticoid receptor antagonists in the CeA suppress drinking specifically in alcohol-dependent rats [38,62,64–66]. In contrast, the anti-stress neuropeptide NPY is decreased in the CeA in alcohol dependence, and intra-CeA infusion of NPY suppresses alcohol drinking specifically in dependent rats [67,68]. We have reported that CRF, similar to alcohol, robustly enhances GABAergic transmission in CeA of rats [38,66,68]. However, NPY and nociceptin decreased presynaptic GABA release in CeA, normalizing the enhanced GABAergic transmission observed in alcohol dependence [68]. Both NPY and nociceptin also block the presynaptic acute alcohol-induced facilitation of CeA GABA release, whereas oxytocin blocked GABA release in dependent rats only. We previously reported that alcohol may act presynaptically through voltage-gated calcium channels to increase action-potential–dependent GABA release in the CeA and that alcohol dependence disrupts this mechanism, shifting alcohol actions to CRF_1_ receptors [52]. This may explain why oxytocin has no effect on eIPSPs in dependent animals if alcohol dependence dysregulates calcium channels and oxytocin effects work through action-potential–induced calcium-dependent mechanisms. Additionally, oxytocin, via action at oxytocin receptors, may interfere with the CRF_1_ receptor-mediated mechanism in dependent rats to blunt the effects of alcohol. Therefore, oxytocin’s actions in the CeA are similar but not identical to anti-stress neuropeptides like NPY [67]. Oxytocin’s actions are particularly complex given the pre- and postsynaptic interactions with alcohol. Future studies will be needed to investigate the functional role of oxytocin in its interactions with other pro-stress and anti-stress systems in the CeA.

One important limitation of the present work is that the studies were conducted exclusively in male rats, especially considering sex-specific oxytocin distribution and behavioral mediation [69,70] and sex differences in alcohol drinking [35,71]. It will be critical to test the effect of oxytocin in female subjects to determine the extent to which the present conclusions can be generalized to females. Second, the behavior and electrophysiology experiments were performed in Wistar and Sprague Dawley rats, respectively. Note that, in general, we find the different rat strains to be better suited to one set of experiments or the other (Wistar for behavior and Sprague-Dawley for electrophysiology). There may be strain differences in the sensitivity of rats to oxytocin treatment (e.g., MacFadyen and colleagues reported that 0.1 mg/kg oxytocin reduced drinking in nondependent Sprague-Dawley rats [18]). However, we included internal controls in behavioral and electrophysiological experiments and have previously shown that baseline CeA synaptic activity and effects of drugs are similar between these strains [72]. As such, we do not find problems conceptually linking the data sets. Lastly, although we tested the oxytocin effects on CeA GABAergic signaling in the presence of a V1a antagonist to rule out the contribution of CeA vasopressin receptors in oxytocin’s effects on CeA GABAergic signaling, the contribution of vasopressin receptors to the observed behavioral effects were not evaluated in the present study. There is evidence suggesting a role of central vasopressin receptors in social and aggressive behaviors, and affective states, including anxiety-like behavior [54]. We would expect that agonism of these receptors by oxytocin would have pro-stress, pro-drinking effects rather than contributing to a reduction in alcohol drinking in dependent rats in this model. Consistent with this hypothesis, Edwards and colleagues reported that V1b antagonism in dependent rats decreased their alcohol drinking [73]. Similar effects have also been reported in Sardinian alcohol-preferring rats [74] and alcohol-dependent humans [75].

In summary, the present study reports that oxytocin via central rather than peripheral action reduced alcohol consumption and motivation for alcohol in an animal model of alcohol dependence. These effects may result from dependence-induced alterations in oxytocin and GABA systems in reward- and stress-related extrahypothalamic brain regions such as the extended amygdala. Intranasal oxytocin administration appears to be advantageous in terms of specificity in reducing alcohol-motivated behavior compared with intraperitoneal administration. Therefore, the present work highlights the oxytocin system as a target for understanding the plasticity of brain stress and anti-stress systems in the etiology of alcohol use disorders. Targeting this system, possibly by intranasal administration, may provide novel pharmaceutical interventions for the treatment of alcohol use disorder.

## Supporting information

S1 Fig Baseline operant responding/reinforcement (FR1) for 10% alcohol (w/v) and water prior to pharmacological testing of intraperitoneal and intranasal oxytocin. (A) Last 8 sessions of operant alcohol self-administration training (FR1) prior to intraperitoneal testing. A 2 × 8 (Group × Session) R.M. ANOVA confirmed a significant effect of Group (*F*_1,18_ = 53.14, *p* < 0.0001). A significant effect of Session (*F*_7, 126_ = 2.14, *p* < 0.05) was also detected, indicating an increase in drinking across sessions. (B) Eight sessions of operant alcohol self-administration training were used to reestablish a baseline of drinking prior to intranasal testing. A 2 × 8 (Group × Session) R.M. ANOVA indicated that the difference between dependent and nondependent rats over 8 sessions was significant (*F*_1, 18_ = 30.58, *p* < 0.0001), and there was a significant main effect of session as behavior stabilized over time (*F*_7, 126_ = 3.397, *p* < 0.01). (C) Water responding/reinforcement during the 8 sessions prior to intraperitoneal testing. A significant decrease in water drinking was observed over sessions (*F*_7, 126_ = 3.54, *p* < 0.01). (D) Water responding/reinforcement was low during the 8 sessions prior to intranasal testing. FR1, fixed-ratio 1; R.M. ANOVA, repeated-measures ANOVA.
(TIF)

S2 Fig Responding/reinforcement on the water lever during pharmacological testing. Water responding remained low relative to alcohol responding/reinforcement in all pharmacological tests. (A) Oxytocin decreased water responding/reinforcement following intraperitoneal administration regardless of group (*F*_4, 72_ = 4.32, *p* < 0.01). Post hoc analyses indicated that responding for water was significantly reduced at the 0.5 mg/kg and 1 mg/kg doses (*p* < 0.01). (B) Water responding/reinforcement was not altered during intranasal oxytocin treatment in either group. (C) Average water consumption was not significantly altered during i.c.v oxytocin administration tests. (D) Water responding/reinforcement was not altered in dependent rats during tests of intranasal oxytocin combined with the peripherally restricted antagonist L-371,257. (E) Intracerebroventricular administration of PF-06655075 significantly lowered water consumption compared with baseline (*F*_2, 12_ = 4.098, *p* < 0.05, post hoc test: *p* < 0.05) but not compared with vehicle. (F) Systemic administration of PF-06655075 significantly lowered water consumption relative to baseline (*F*_2, 16_ = 3.948, *p* < 0.05, post hoc test: *p* < 0.05), whereas its vehicle did not. i.c.v, intracerebroventricular.
(TIF)

S3 Fig Effect of oxytocin on spike frequency. Spike frequency at 2 injected currents under baseline conditions for neurons from nondependent (200 pA: 1.55 ± 0.46 spikes, 400 pA: 4.18 ± 0.57 spikes) and alcohol-dependent (200 pA: 2.47 ± 0.55 spikes, 400 pA: 4.73 ± 0.91 spikes) rats. Oxytocin (500 nM) did not alter spike frequency at either current level for nondependent (200 pA: 1.91 ± 0.46 spikes, 400 pA: 4.91 ± 0.58 spikes) or dependent (200 pA: 1.87 ± 0.73 spikes, 400 pA: 4.87 ± 1.10 spikes) neurons.
(TIF)

S4 Fig I/O baseline eIPSP amplitude relationship for nondependent and alcohol-dependent animals. eIPSP I/O curves generated by 5 equivalent normalized stimulus intensities between nondependent (3.7 ± 0.3, 7.0 ± 0.6, 9.7 ± 0.7, 12.2 ± 0.8, and 14.7 ± 0.9 mV) and dependent (3.7 ± 0.3, 6.8 ± 0.5, 9.9 ± 0.7, 12.8 ± 0.8, and 14.9 ± 0.9 mV) animals. eIPSP, evoked inhibitory postsynaptic potential; I/O, input–output.
(TIF)

S5 Fig Effect of alcohol and TMA on eIPSPs. Alcohol (44 mM) increased evoked GABA responses in neurons from nondependent and dependent rats, an effect unaffected by the vasopressin 1A receptor antagonist TMA. eIPSP, evoked inhibitory postsynaptic potential; TMA, (d(CH2)5,Tyr(Me)2,Arg8)-Vasopressin.
(TIF)

S6 Fig Effect of TMA on sIPSCs. Baseline GABA_A_-mediated sIPSC frequency, amplitude, and kinetic measurements (rise and decay time) are unaffected by vasopressin 1A receptor antagonist (TMA) application in CeA neurons from nondependent and alcohol dependent rats. CeA, central nucleus of the amygdala; sIPSC, spontaneous inhibitory postsynaptic current; TMA, (d(CH2)5,Tyr(Me)2,Arg8)-Vasopressin.
(TIF)

S7 Fig Effect of OTA on sIPSCs. The oxytocin receptor antagonist OTA did not affect sIPSC frequency, amplitude, or kinetics in CeA neurons from dependent animals. CeA, central nucleus of the amygdala; OTA, desGly-NH2-d(CH2)5[D-Tyr2,Thr4]OVT; sIPSC, spontaneous inhibitory postsynaptic current.
(TIF)

S8 Fig Effect of OTA on eIPSPs. The oxytocin receptor antagonist OTA did not affect eIPSP amplitude but blocked oxytocin induced decreases in amplitude and restored ethanol induced increases in amplitude in CeA neurons from nondependent animals. CeA, central nucleus of the amygdala; eIPSP, evoked inhibitory postsynaptic potential; OTA, desGly-NH2-d(CH2)5[D-Tyr2,Thr4]OVT.
(TIF)

S1 Text This file contains supplemental methods and results. (DOCX)

S1 Data This file contains the raw data presented in figures in the main manuscript (Figs 1–5) and supplemental figures (S1–S8 Figs). (XLSX)

## Abbreviations

aCSF: artificial cerebrospinal fluid
CeA: central nucleus of the amygdala
CRF: corticotropin-releasing factor
DL-AP5: DL-2-amino-5-phosphonovalerate
DNQX: 6,7-dinitroquinoxaline-2,3-dione
eIPSP: evoked inhibitory postsynaptic potential
FR1: fixed-ratio 1
I/O: input–output
IACUC: Institutional Animal Care and Use Committee
mIPSC: miniature inhibitory postsynaptic current
NPY: neuropeptide Y
OTA: desGly-NH2-d(CH2)5[D-Tyr2,Thr4]OVT
PPR: paired-pulse ratio
PR: progressive ratio
R.M. ANOVA: repeated-measures ANOVA
rPOD: rat Precision Olfactory Device
sIPSC: spontaneous inhibitory postsynaptic current
TGOT: (Thr⁴,Gly⁷)-Oxytocin
TMA: (d(CH2)5,Tyr(Me)2,Arg8)-Vasopressin
TTX: Tetrodotoxin
