# Supplementary figures and images for: Oxytocin blocks enhanced motivation for alcohol in alcohol dependence and blocks alcohol effects on GABAergic transmission in the central amygdala
Source: PLoS Biol. 2019 Apr 16;17(4):e2006421. doi: 10.1371/journal.pbio.2006421 (PMC6467366; doi:10.1371/journal.pbio.2006421)

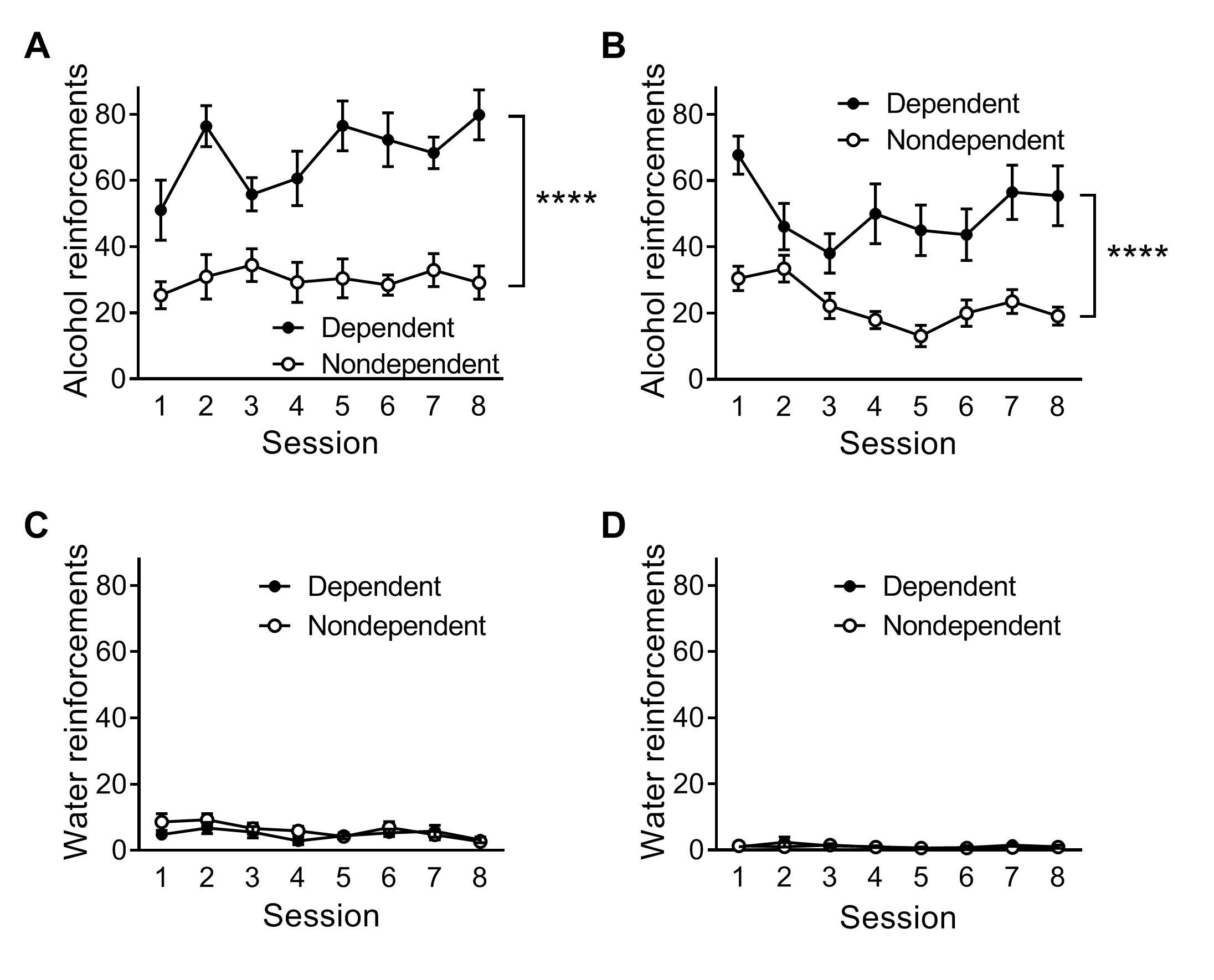

Supplement: S1 Fig — (A) Last 8 sessions of operant alcohol self-administration training (FR1) prior to intraperitoneal testing. A 2 × 8 (Group × Session) R.M. ANOVA confirmed a significant effect of Group (F1,18 = 53.14, p < 0.0001). A significant effect of Session (F7, 126 = 2.14, p < 0.05) was also detected, indicating an increase in drinking across sessions. (B) Eight sessions of operant alcohol self-administration training were used to reestablish a baseline of drinking prior to intranasal testing. A 2 × 8 (Group × Session) R.M. ANOVA indicated that the difference between dependent and nondependent rats over 8 sessions was significant (F1, 18 = 30.58, p < 0.0001), and there was a significant main effect of session as behavior stabilized over time (F7, 126 = 3.397, p < 0.01). (C) Water responding/reinforcement during the 8 sessions prior to intraperitoneal testing. A significant decrease in water drinking was observed over sessions (F7, 126 = 3.54, p < 0.01). (D) Water responding/reinforcement was low during the 8 sessions prior to intranasal testing. FR1, fixed-ratio 1; R.M. ANOVA, repeated-measures ANOVA. (TIF) [file pbio.2006421.s001.tif]

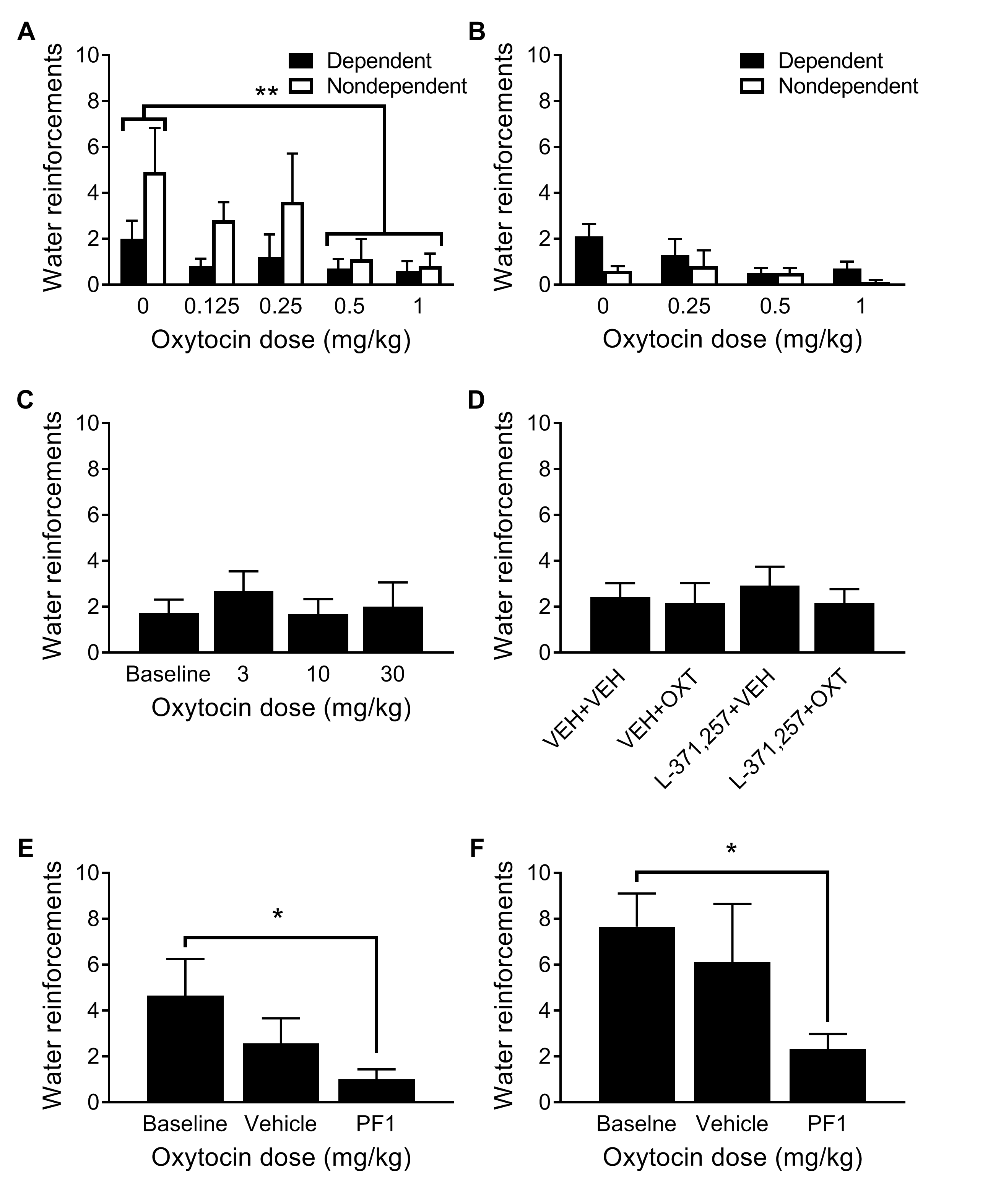

Supplement: S2 Fig — Water responding remained low relative to alcohol responding/reinforcement in all pharmacological tests. (A) Oxytocin decreased water responding/reinforcement following intraperitoneal administration regardless of group (F4, 72 = 4.32, p < 0.01). Post hoc analyses indicated that responding for water was significantly reduced at the 0.5 mg/kg and 1 mg/kg doses (p < 0.01). (B) Water responding/reinforcement was not altered during intranasal oxytocin treatment in either group. (C) Average water consumption was not significantly altered during i.c.v oxytocin administration tests. (D) Water responding/reinforcement was not altered in dependent rats during tests of intranasal oxytocin combined with the peripherally restricted antagonist L-371,257. (E) Intracerebroventricular administration of PF-06655075 significantly lowered water consumption compared with baseline (F2, 12 = 4.098, p < 0.05, post hoc test: p < 0.05) but not compared with vehicle. (F) Systemic administration of PF-06655075 significantly lowered water consumption relative to baseline (F2, 16 = 3.948, p < 0.05, post hoc test: p < 0.05), whereas its vehicle did not. i.c.v, intracerebroventricular. (TIF) [file pbio.2006421.s002.tif]

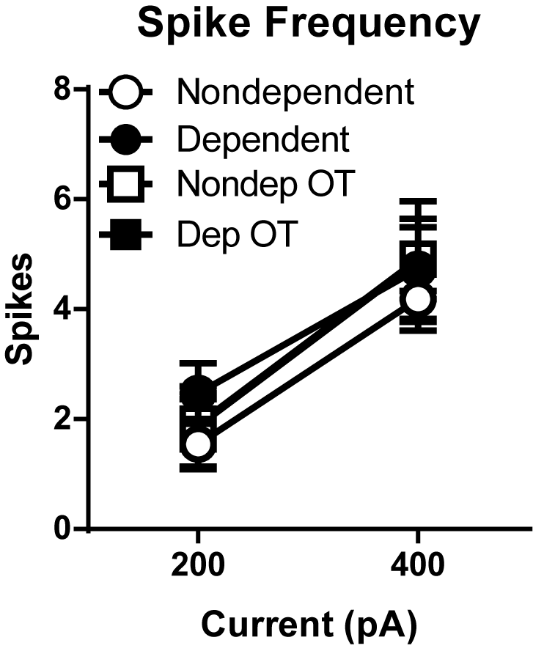

Supplement: S3 Fig — Spike frequency at 2 injected currents under baseline conditions for neurons from nondependent (200 pA: 1.55 ± 0.46 spikes, 400 pA: 4.18 ± 0.57 spikes) and alcohol-dependent (200 pA: 2.47 ± 0.55 spikes, 400 pA: 4.73 ± 0.91 spikes) rats. Oxytocin (500 nM) did not alter spike frequency at either current level for nondependent (200 pA: 1.91 ± 0.46 spikes, 400 pA: 4.91 ± 0.58 spikes) or dependent (200 pA: 1.87 ± 0.73 spikes, 400 pA: 4.87 ± 1.10 spikes) neurons. (TIF) [file pbio.2006421.s003.tif]

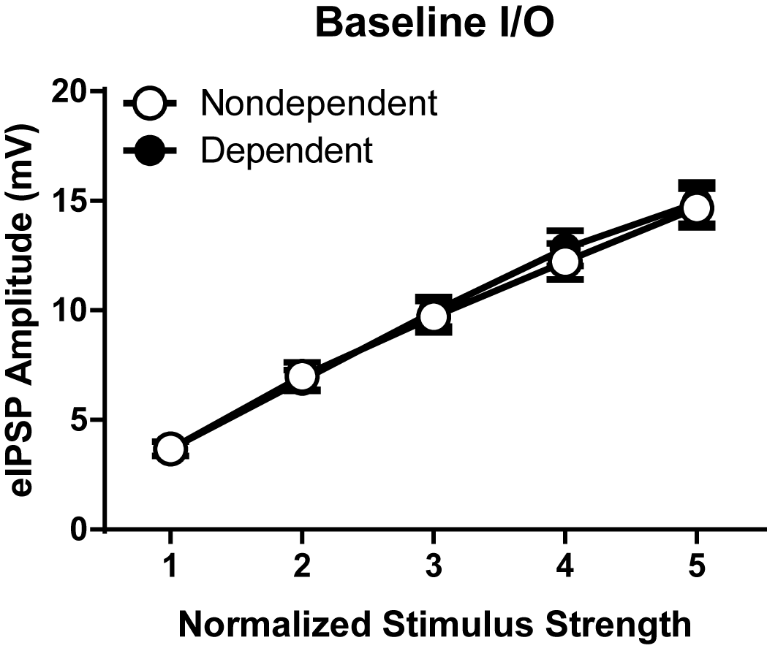

Supplement: S4 Fig — eIPSP I/O curves generated by 5 equivalent normalized stimulus intensities between nondependent (3.7 ± 0.3, 7.0 ± 0.6, 9.7 ± 0.7, 12.2 ± 0.8, and 14.7 ± 0.9 mV) and dependent (3.7 ± 0.3, 6.8 ± 0.5, 9.9 ± 0.7, 12.8 ± 0.8, and 14.9 ± 0.9 mV) animals. eIPSP, evoked inhibitory postsynaptic potential; I/O, input–output. (TIF) [file pbio.2006421.s004.tif]

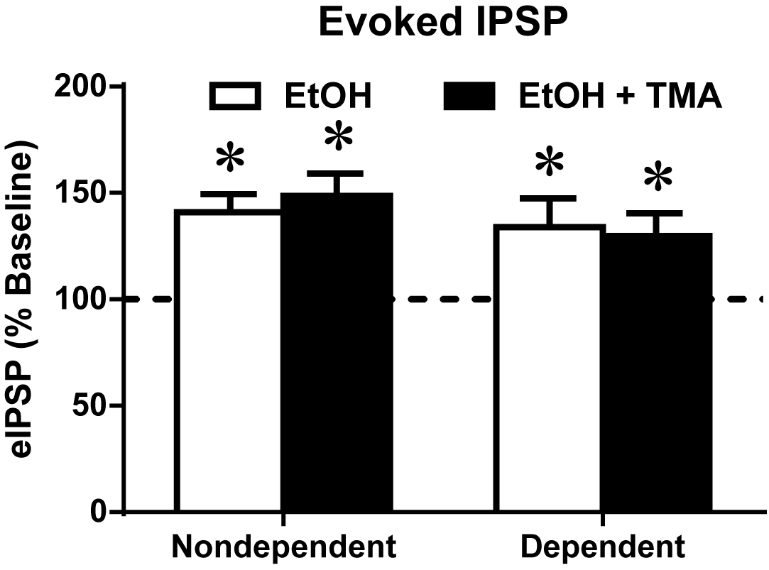

Supplement: S5 Fig — Alcohol (44 mM) increased evoked GABA responses in neurons from nondependent and dependent rats, an effect unaffected by the vasopressin 1A receptor antagonist TMA. eIPSP, evoked inhibitory postsynaptic potential; TMA, (d(CH2)5,Tyr(Me)2,Arg8)-Vasopressin. (TIF) [file pbio.2006421.s005.tif]

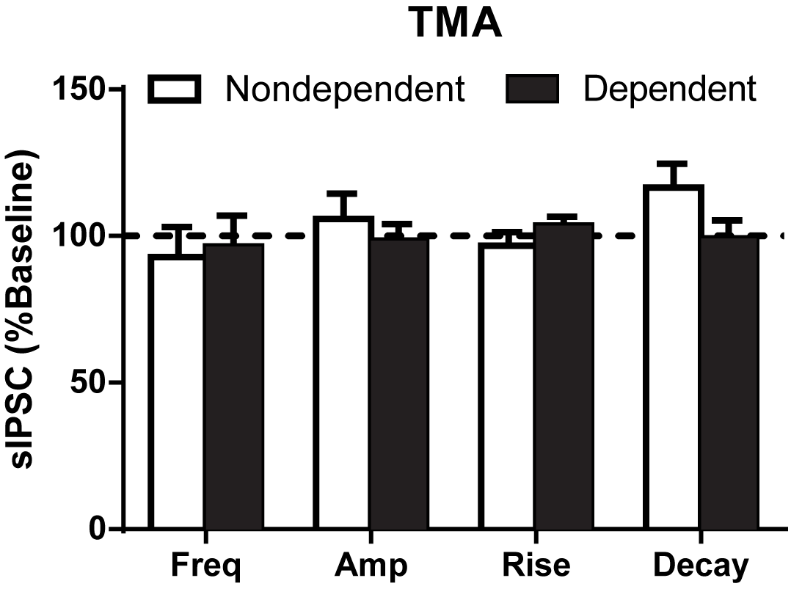

Supplement: S6 Fig — Baseline GABAA-mediated sIPSC frequency, amplitude, and kinetic measurements (rise and decay time) are unaffected by vasopressin 1A receptor antagonist (TMA) application in CeA neurons from nondependent and alcohol dependent rats. CeA, central nucleus of the amygdala; sIPSC, spontaneous inhibitory postsynaptic current; TMA, (d(CH2)5,Tyr(Me)2,Arg8)-Vasopressin. (TIF) [file pbio.2006421.s006.tif]

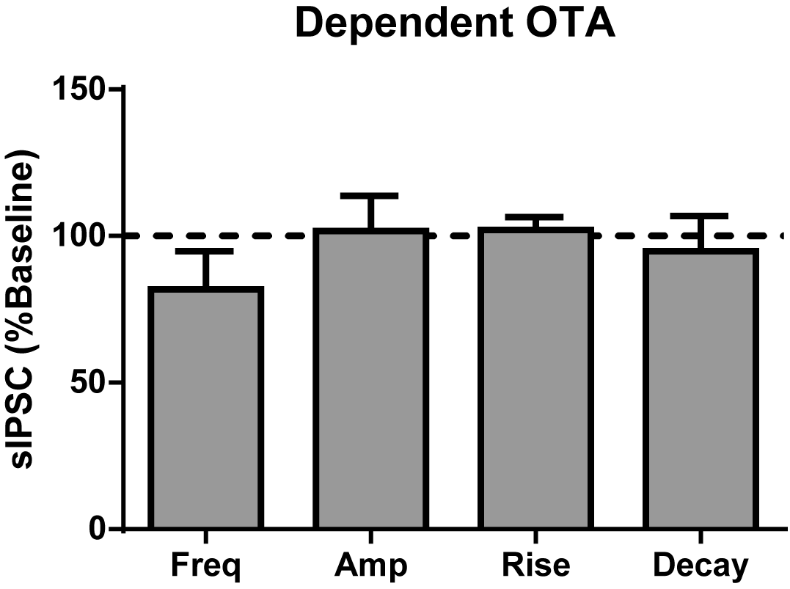

Supplement: S7 Fig — The oxytocin receptor antagonist OTA did not affect sIPSC frequency, amplitude, or kinetics in CeA neurons from dependent animals. CeA, central nucleus of the amygdala; OTA, desGly-NH2-d(CH2)5[D-Tyr2,Thr4]OVT; sIPSC, spontaneous inhibitory postsynaptic current. (TIF) [file pbio.2006421.s007.tif]

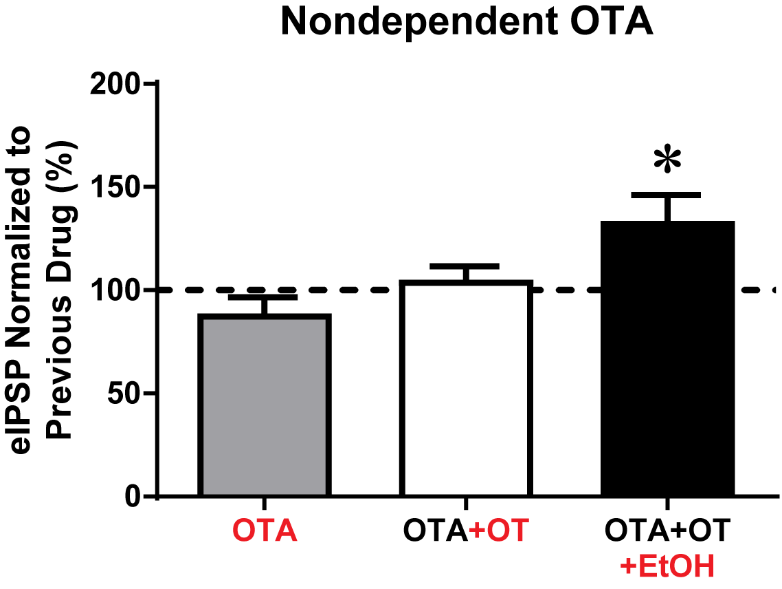

Supplement: S8 Fig — The oxytocin receptor antagonist OTA did not affect eIPSP amplitude but blocked oxytocin induced decreases in amplitude and restored ethanol induced increases in amplitude in CeA neurons from nondependent animals. CeA, central nucleus of the amygdala; eIPSP, evoked inhibitory postsynaptic potential; OTA, desGly-NH2-d(CH2)5[D-Tyr2,Thr4]OVT. (TIF) [file pbio.2006421.s008.tif]
