# Supplementary material for: Oxytocin blocks enhanced motivation for alcohol in alcohol dependence and blocks alcohol effects on GABAergic transmission in the central amygdala
Source: PLoS Biol. 2019 Apr 16;17(4):e2006421. doi: 10.1371/journal.pbio.2006421 (PMC6467366; doi:10.1371/journal.pbio.2006421)
Supplement: S1 Text — (DOCX) [file pbio.2006421.s009.docx]

**Oxytocin blocks compulsive-like alcohol drinking and blocks alcohol effects on GABAergic transmission in the central amygdala**

Brendan J. Tunstall, Dean Kirson, Lia J. Zallar, Sam A McConnell, Janaina C.M. Vendruscolo, Chelsea P. Ho, Christopher S. Oleata, Sophia Khom, Maurice Manning, Mary R. Lee, Lorenzo Leggio, George F. Koob, Marisa Roberto & Leandro F. Vendruscolo

***Supplemental Information***

**Supplemental Methods**

*Effect of intraperitoneal and intranasal oxytocin on open field locomotion and grooming, motor coordination, and consumption of nonalcoholic palatable solutions*

A separate cohort of dependent (*n* = 6) and nondependent (*n* = 6) rats was used to test the effects of oxytocin on spontaneous locomotion in an open field (40 cm X 40 cm arena, 5-min test). Rats were tested following administration of intraperitoneal saline and oxytocin, as well as intranasal saline and oxytocin in a Latin-square design.

A separate cohort of dependent (*n* = 5) and nondependent (*n* = 7) rats were used to test the effects of oxytocin on motor coordination on a rotarod (accelerating from 4 to 40 RPM over a 5-min test). Counterbalanced intraperitoneal saline *versus* oxytocin tests occurred first, followed by counterbalanced intranasal saline *versus* oxytocin tests.

Because alcohol has caloric and gustatory reinforcing properties, separate cohorts of rats were used to test the effect of oxytocin on the intake of nonalcoholic palatable solutions. Rats (*n*=8) were trained in 11, 30-min sessions (5 days per week) to self-administer (FR1) 0.1% saccharin (w/v in water; sweet/noncaloric). A separate group (*n*=7) was trained for 5, 30-min sessions to self-administer (FR1) 5% maltodextrin (w/v in water; nonsweet/caloric). Rats in both groups were tested in regular self-administration sessions, preceded by administration of intraperitoneal saline *versus* oxytocin, as well as intranasal saline *versus* oxytocin, in a Latin-square design.

*Central versus peripheral mediation of oxytocin’s effect on alcohol intake*

Three separate cohorts of alcohol dependent rats were used in four experiments designed to test for the possible central *versus* peripheral mediation of oxytocin’s effect on alcohol intake (central oxytocin administration: *n*=6; peripheral oxytocin receptor agonist: *n*= 9; peripheral oxytocin receptor antagonist combined with intransal oxytocin administration: *n*=12). A subset of the group used for testing the peripheral oxytocin receptor antagonist combined with intranasal oxytocin administration was reused to test the effect of central administration of PF-06655075, which is an oxytocin receptor agonist that does not cross the blood-brain barrier (central oxytocin receptor agonist: *n*=7). All rats were trained to self-administer alcohol and made alcohol dependent, as described in the main manuscript. As during testing of intraperitoneal and intranasal oxytocin, operant alcohol self-administration sessions including pharmacological tests were conducted 2-3 sessions per week (never on consecutive days to minimize potential carry-over effects) during the 10 h OFF period, 6-8 h into withdrawal. To test the effect of central oxytocin administration, six rats underwent surgical implantation of a 26-gauge intracranial cannulae directed at the lateral ventricle (with the skull flat; -0.8 A/P, +/- 1.6 M/L, -3.0 D/V from dura). Correct placement was verified during the surgery by gravity infusion of saline through a 33-gauge injector projecting 1 mm beyond the cannulae. After a week recovery, stable baseline responding was reestablished. A 10 µl Hamilton glass syringe was used to administer ascending doses of oxytocin (3, 10, and 30 µg, dissolved in 5 µl of saline) 30 min prior to self-administration sessions. These rats were repurposed after previously being used to test ICV injection of a compound that was found to be behaviorally inactive. Nonetheless, to avoid any possible carry-over effects, rats were given a washout period of five days followed by two baseline FR1 sessions without any pre-treatment before the test phase began. A baseline FR1 session of alcohol self-administration was also given between each test session, during which no treatment was administered.

To further test the central *versus* peripheral contribution of oxytocin to alcohol drinking, a long-acting, large molecule, non brain-penetrant, oxytocin receptor agonist (PF-06655075) was used. Because the central oxytocin receptor agonist cohort of rats was previously used to test the peripheral oxytocin receptor antagonist combined with intranasal oxytocin administration, two FR1 session of alcohol self-administration in the absence of any treatment were conducted to reestablish a baseline before rats underwent surgical implantation of a cannula directed at the lateral ventricule, as described for the central oxytocin administration cohort. The rats were allowed to recover from surgery and then a baseline FR1 session of alcohol self-administration was given to confirm that rats maintained stable responding. The dose of 30 µg of PF-06655075 was selected to match the highest dose of oxytocin administered intracerebroventricularly, that significantly reduced alcohol drinking in alcohol-dependent rats. A 10 µl Hamilton glass syringe was used to administer PF-06655075 and its vehicle (10 µl consisting of 5% v/v dimethyl sulfoxide, 5% v/v polyethylene glycol 300, and 90% v/v saline). The first alcohol self-administration test session was conducted with vehicle treatment and the second test session with 30 µg of PF-06655075; the pretreatment time was 30 min. To avoid any possible carry-over effects, a baseline FR1 session of alcohol self-administration was also given between each test session, during which no treatment was administered.

In the peripheral oxytocin receptor agonist cohort, vehicle or 1 mg/kg of the agonist (4 ml/kg) were administered subcutaneously 1 h prior to FR1 alcohol self-administration sessions. The pretreatment time was 1 h. In selecting the peripheral dose of the agonist, it was considered that due to the high plasma protein binding of PF-06655075, the unbound concentrations are significantly lower than those of oxytocin. For example, as shown in Fig. 2B of Modi et al. [1], following subcutaneous administration at 1 mg/kg, the total Cmax values were 331 nM for PF-06655075 and 191 nM for oxytocin. However, when the plasma protein binding of each compound is considered, the unbound concentrations are very different. The unbound Cmax for PF-06655075 was determined to be 0.6 nM *versus* 191 nM for oxytocin. As mentioned in Modi et al. [1], following the free drug hypothesis, it is presumed that only unbound concentrations of the compound are pharmacologically active; thus, the concentrations tested for PF-06655075 are in a significantly lower range in comparison to oxytocin to elicit pharmacological activity. However, considering unbound plasma concentration with the oxytocin receptor binding Ki, both compounds had unbound concentrations in excess of their respective Ki values with a significantly higher excess for oxytocin (PF-06655075 unbound plasma/Ki = 16x; oxytocin unbound plasma/Ki = 398x; set using a Ki = 0.037 nM for PF-06655075 and Ki = 0.48 nM for oxytocin based on Modi et al. [1]). If this is taken further into calculated receptor occupancy, at Cmax 1 mg/kg, PF-06655075 would be estimated to produce approximately 94.2% of receptor occupancy and 1 mg/kg oxytocin would be estimated to produce 99.7% of receptor occupancy. Because these are both agonists, both of these receptor occupancy values would be expected to produce a pharmacological effect. On-target pharmacological effects would be expected to be similar (for clarity, the calculated receptor occupancy is for plasma and is based on the unbound plasma Cmax and oxytocin receptor binding Ki values). Further supporting the notion that this dose of PF-06655075 is sufficient to recapitulate the peripheral actions of oxytocin, the dose of PF-06655075 administered (1 mg/kg) is four times higher than the intraperitoneal dose of oxytocin that blocked dependence-induced drinking in the present study (0.25 mg/kg). Additionally, the dose and pre-treatment time used have been demonstrated to produce behavioral effects [1].

*Electrophysiological Slice Preparation*

Alcohol dependent rats (*n*=37) were exposed to the same schedule of chronic, intermittent alcohol vapor exposure as in behavioral experiments for 5-7 weeks. Blood alcohol levels were measured weekly by tail-bleeding (the mean blood alcohol level upon euthanasia was 175.8 ± 7.5 mg/dL). Rats were anesthetized with isoflurane (3-5%) followed by rapid decapitation and immediate removal of the brain into an ice-cold high sucrose cutting solution (sucrose 206 mM; KCl 2.5 mM; CaCl_2_ 0.5 mM; MgCl_2_ 7 mM; NaH_2_PO_4_ 1.2 mM; NaHCO_3_ 26 mM; glucose 5 mM; HEPES 5 mM; pH 7.4). For dependent rats, euthanasia occurred 15-30 min prior to the end of their final alcohol vapor exposure and slice preparation occurred in alcohol-free solutions. As a result, the electrophysiology recordings were obtained from neurons undergoing acute withdrawal (1-8 h). CeA coronal slices (300-400 µm) were incubated in an interface configuration for 30 min to 1 h, and then submerged and continuously superfused (flow rate of 2-4 ml/min) with 95% O_2_/5% CO_2_ equilibrated artificial cerebrospinal fluid (aCSF) of the following composition: NaCl 130 mM; KCl 3.5 mM; NaH_2_PO_4_ 1.25 mM; MgSO_4_·7H_2_O 1.5 mM; CaCl_2_ 2.0 mM; NaHCO_3_, 24 mM; glucose 10 mM. Recordings were performed in neurons from the medial subdivision of the CeA, and each experimental group contained neurons from a minimum of three animals. GABAergic activity was pharmacologically isolated with 20 μM DNQX (to block AMPA receptors), 30 μM DL-AP5 (to block NMDA receptors), and 1 μM CGP 55845A (to block GABA-B receptors). All drugs were applied by bath superfusion.

*Intracellular Recordings of Evoked IPSPs*

We recorded with sharp micropipettes filled with 3M KCl using discontinuous current-clamp mode in the medial subdivision of the CeA. Neurons were held near their resting membrane potential (-82.4±0.8 mV). We evoked GABAergic inhibitory postsynaptic potentials (eIPSPs) by stimulating locally within the CeA through a bipolar stimulating electrode. We performed an input–output (I/O) protocol consisting of a range of 5 current stimulations, starting at the threshold current required to elicit an eIPSP, up to the strength required to elicit the maximum subthreshold amplitude. These stimulus strengths were maintained throughout the duration of the experiment. We also performed paired pulse ratio (PPR), where two equal intensity stimuli are applied in rapid succession, and the ratio is calculated as the amplitude of the second postsynaptic potential over that of the first postsynaptic potential. PPR was performed at the stimulus intensity giving ~50% of the maximal amplitude determined in the I/O protocol. In general, a drug-induced increase in PPR suggests a decrease in presynaptic neurotransmitter (GABA) release, and vice versa. All measures were performed prior to (baseline) and during drug application.

*Whole-Cell Patch Clamp Recording of spontaneous IPSC*

We recorded GABAergic spontaneous and miniature inhibitory postsynaptic currents (sIPSCs/mIPSCs) from neurons visualized in brain slices using infrared differential interference contrast (IR-DIC) optics and CCD cameras (QImaging, Surrey, BC, Canada). Whole-cell voltage-clamp recordings were acquired with a Multiclamp 700B amplifier (Molecular Devices) at 50 kHz and low-pass filtered at 10 kHz, digitized (Digidata 1440A; Molecular Devices), and stored on a PC using pClamp 10 software (Axon Instruments). Patch pipettes (3-6 MΩ) were pulled from borosilicate glass (Warner Instruments, Hamden, CT) and filled with an internal solution composed of (in mM): 145 KCl; 0.5 EGTA; 2 MgCl2; 10 HEPES; 2 Na-ATP; 0.2 Na-GTP. All voltage clamp recordings were performed in a gap-free acquisition mode, with cells clamped at –60 mV for the duration of the recordings. In all experiments, cells with a series resistance greater than 25 MΩ were excluded from analysis, and series resistance was continuously monitored during gap free recording with a 10-mV pulse. Cells where series resistance changed more than 25% during the course of the experiment were excluded from analysis. All measures were performed prior to (baseline) and during drug application. Frequency, amplitude, and kinetics of sIPSCs were analyzed. Averages of sIPSC characteristics were based on a minimum time interval of 3 min and a minimum of 50 events.

**Supplemental Results**

*Effect of intraperitoneal and intranasal oxytocin on open field locomotion, rotarod motor coordination, and consumption of nonalcoholic palatable solutions*

Responding on the water lever was very low relative to the available reinforcement alternative in groups trained to respond for saccharin (average responding on last 3 training sessions for saccharin = 138.0 ± 14.7, for water = 4.0 ± 0.6) or for maltodextrin (average responding on last 3 training sessions for maltodextrin = 156.1 ± 10.8, for water = 6.2 ± 1.0). Responding on the water lever remained low across saccharin (water lever presses = 0.8 ± 0.2) and maltodextrin (water lever presses = 3.3 ± 0.9) test sessions compared to the available reinforcement alternative (see Fig. 2C). Neither intraperitoneal nor intranasal oxytocin altered water intake significantly compared to vehicle during saccharin or maltodextrin test sessions (*t*’s <1.4, *p*’s>0.2).

**Supplemental References**

1. Modi ME, Majchrzak MJ, Fonseca KR, Doran A, Osgood S, Vanase-Frawley M, et al. Peripheral Administration of a Long-Acting Peptide Oxytocin Receptor Agonist Inhibits Fear-Induced Freezing. J Pharmacol Exp Ther. 2016;358(2):164-72. Epub 2016/05/25. doi: 10.1124/jpet.116.232702. PubMed PMID: 27217590; PubMed Central PMCID: PMCPMC4959095.
